# Reconstruction of the rRNA Sequences of LUCA, with Bioinformatic Implication of the Local Similarities Shared by Them

**DOI:** 10.3390/biology11060837

**Published:** 2022-05-29

**Authors:** Yu Men, Guoliang Lu, Yanhui Wang, Jinzhong Lin, Qiang Xie

**Affiliations:** 1 State Key Laboratory of Biocontrol, School of Life Sciences, Sun Yat-sen University, Guangzhou 510275, China; meny@mail2.sysu.edu.cn (Y.M.); wangyanh3@mail.sysu.edu.cn (Y.W.); 2 State Key Laboratory of Genetic Engineering, School of Life Sciences, Zhongshan Hospital, Fudan University, Shanghai 200438, China; luguoliang@fudan.edu.cn (G.L.); linjinzhong@fudan.edu.cn (J.L.)

**Keywords:** ribosomal RNA, structure, last universal common ancestor, ancestral sequence, ribosome

## Abstract

**Simple Summary:**

In order to explore the origin of 16S, 5S, and 23S ribosomal RNAs in novel views and methods, full lengths of the three rRNA sequences of the last universal common ancestor were reconstructed for the first time. Within these sequences, repeat short fragments or local self-similarities were shared. Moreover, these short fragments were conserved, clustered around the functional center of ribosome, and contained nearly all types of functional sites of ribosome. These results indicated a possibility that short fragments may act as component elements or parts of them in the origin of rRNAs, which can be practically tested by simulating experiments in the future.

**Abstract:**

The theory of the RNA world, especially with the catalytic capability of RNA, provides a reasonable framework explaining the evolution of molecular genetics system before the scenario of the central dogma. However, it remains a challenge to deduce the origin mechanism of rRNAs. Here we reconstructed the phylogenetic relationships of archaea and bacteria with bootstrap values of most nodes, especially the deep ones, higher than 90%. Based on the well-resolved tree, the full lengths of 16S, 5S, and 23S rRNA sequences of the last universal common ancestor (LUCA) were reconstructed for the first time. The potential similarities shared by the three ancestral rRNA sequences were further explored by searching for repeat short fragments in the level of purine–pyrimidine (RY) with certain lengths and arrangements. With the lengths ranging from 2 to 14, functional short fragments could be found in the three RNAs. As a representative, a set with a total of 75 short fragments of 11 nucleotides in length can recover all types of the known functional sites of ribosomes in a most concise manner. The 75 short fragments cluster around the functional center of the ribosome, among which 18 of them are highly conserved across five or six kingdoms and still contain all types of known functional sites except one. Alternatively, according to the strategy using the level of AUGC instead of RY, a similar pattern can be recovered. Such results indicate the local similarities shared by 16S, 5S, and 23S rRNAs and thus suggest a possible general mechanism in the formation of the LUCA rRNAs.

## 1. Introduction

The RNA world was first proposed in the 1980s [1], in which RNA served as both the informational and the functional molecule [2,3]. In other words, RNA constituted a self-replicating system [3] about 4 billion years ago, and RNAs with different roles guaranteed their self-sustainability. This theory together with the catalytic capability of RNA [4,5] solved the chicken-or-egg dilemma in which protein and DNA emerged later. Both the origin simulated by Urey–Miller experiments [6,7] and evolution before and after cellular life has been witnessed by one of the most ancient molecules, the ribosome. Ribosomal RNAs are complex, large, and highly conserved, which include 16S rRNA that serves as a component of the small subunit (SSU), and 5S and 23S rRNAs that serve as components of the large subunit (LSU) of the ribosome. The close cooperation between the three rRNA molecules, which vary greatly in length, makes their origins haunt researchers.

The origin and evolution of ribosomes have been investigated based on the structures of extant species in numerous studies. Ribosomes originated in the RNA world and increased in size over time. At the time of LUCA, the ribosome had largely formed [8]. The relative ages of 12 generations of acquired elements in *Escherichia coli* 23S rRNA were inferred based on the A-minor interactions [9]. Later, six phases of the accretion model leading to the LUCA ribosome were proposed based on insertion fingerprints [10]. Considering that the self-replicated fragments could been preserved in their descendants [3] and thus increased in number in the RNA world, all fragments may float in the original soup and the fragments with larger proportions would be more likely to take part in or contribute to the formation of rRNA chains. The relics of these fragments might hide in the rRNA sequences of the LUCA. Thus, we explored the origin and evolution of rRNAs in the novel views and methods of phylogenetics and ancestral state reconstruction. We reconstructed the rRNA sequences of LUCA and searched for repeat short fragments among them.

**Figure 1:**
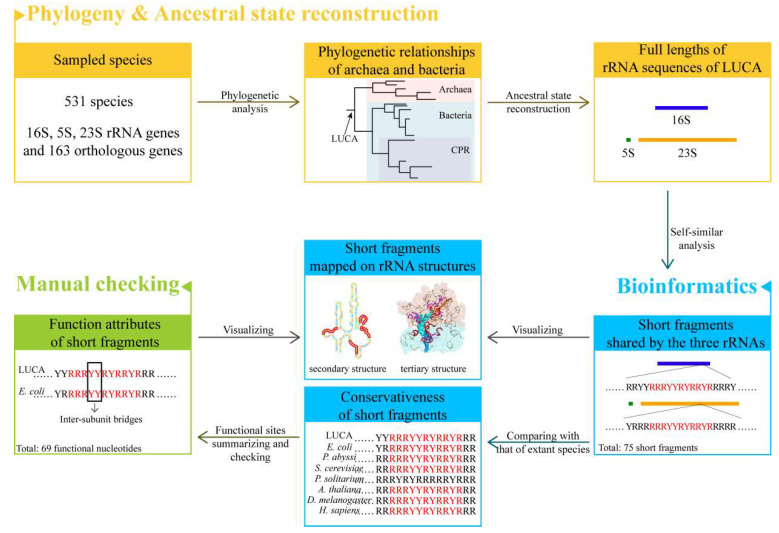
Flow chart overview.

We reconstructed the phylogenetic relationships of archaea and bacteria and thereafter the full length of three rRNA sequences of LUCA. The ancestral sequences were analyzed in the approach of self-similarity (Figure 1). Self-similarity here was defined to be the existence of similar short fragments among the three rRNA sequences. The analysis of self-similarity was conducted by searching for the short repeat fragments with the same arrangements of RY or AUGC shared by ancestral 16S, 5S, and 23S rRNAs. Because the two strategies (AUGC level and RY level) may provide complemented information, both of them were taken into consideration while searching for repeat short fragments. Compared to the AUGC level, searching for short fragments in the RY level gave more accommodation to the transversion in rRNA chains during the evolution of the LUCA ribosome, and more information could be found. However, to obtain more comprehensive results, both strategies were used to explore the potential similarities of the three rRNAs.

Short fragments shared by the three ancestral rRNA sequences were searched and filtered, and their conservativeness was investigated by comparison with their orthologous rRNA sequences of species that belong to six kingdoms. Extraordinarily, some short fragments were highly conserved across five or six kingdoms. The functional attributes of these short fragments were further checked with the known functional sites of ribosome summarized by the structural studies of *E. coli* and other extant species.

## 2. Materials and Methods

### 2.1. Taxon Sampling

A total of 531 species belonging to 153 phyla and candidate phyla of archaea and bacteria was sampled, including 108 species in 18 archaeal phyla and 423 species in 135 bacterial phyla (Table S1). The representatives covered almost all phyla recorded in the NCBI database (www.ncbi.nlm.nih.gov, accessed on 7 January 2021) and EzBioCloud (https://help.ezbiocloud.net/ezbiocloud-16s-database/, accessed on 1 April 2019) [11], and at least 3 species were sampled in each archaeal and bacterial phylum. For the phyla containing less than 3 species whose genomes or 16S rRNA genes were available, all species were sampled.

### 2.2. Phylogenetic Analysis

Orthograph (Version 0.6.3, Petersen Malte, Bonn, Germany) [12] was used to map candidate orthologous genes from the genomes of representatives to a target orthologous gene set. The genomes of these sampled species were downloaded from the NCBI database. To generate a set of orthologous genes, we selected the genes in the genomes of the following eight reference species: *Cenarchaeum symbiosum* (Archaea: Thaumarchaeota), *Ignisphaera aggregans* (Archaea: Crenarchaeota), *Methanothermus fervidus* (Archaea: Euryarchaeota), *Bacillus cereus* (Bacteria: Firmicutes), *Crinalium epipsammum* (Bacteria: Cyanobacteria), *Fluviicola taffensis* (Bacteria: Bacteroidetes), *Rubrobacter radiotolerans* (Bacteria: Actinobacteria), and *Escherichia coli* (Bacteria: Proteobacteria). Only the genes presenting in at least two genomes of the 8 reference species were selected, which resulted in 989 protein coding genes. Finally, the genomes of all species were searched for these 989 target genes with the best-reciprocal hit (BRH) criterion, and the results were summarized to generate 989 preliminary amino acid data sets. We combined these 989 preliminary orthologous gene sets together with the 381 data sets of the article, which were justified as informative in the phylogenetic analyses of bacteria and archaea [13], and the same genes were deleted. We selected the gene sets with more than half representatives, leaving 163 amino acid data sets. Moreover, the sequences of 16S, 5S, and 23S rRNAs were downloaded from the NCBI database (https://www.ncbi.nlm.nih.gov/, accessed on 10 January 2020) and the 16S database from the website of EzBioCloud (https://help.ezbiocloud.net/ezbiocloud-16s-database/, accessed on 1 April 2019) [11].

All of the 166 gene sets were aligned individually with MAFFT (Version 7.490, Katoh Kazutaka, Osaka, Japan) [14]. Ambiguously aligned sites were removed using GBlocks (Version 0.91b, Talavera Gerard, Barcelona, Spain) [15]. Then all the gene sets were concatenated using Sequence Matrix (Version 1.7.8, Vaidya Gaurav, Singapore) [16] to generate a final concatenated matrix. The gene sets of 163 protein coding genes, 3 rRNA genes, and the concatenated matrix were deposited on DataOpen (http://dataopen.info/home/datafile/index/id/254, uploaded on 3 May 2022), and provided as Supplementary Materials (Data S1). The order and the range of the rDNAs and of the proteins in the concatenated matrix were provided as Data S2. The best partitioning schemes and their best substitution models were identified using IQ-TREE (Version 1.6.10, Lam-Tung Nguyen, Vienna, Austria) [17], and the results calculated by IQ-TREE were provided as Data S3.

Phylogenetic analysis was performed by RAxML (Version 8, Stamatakis Alexandros, Heidelberg, Germany) [18] using a rapid bootstrap algorithm with 200 replicates. RAxML supports the analysis of heterogeneous data types, so we concatenated the alignment of rDNAs and that of amino acids into a single matrix. According to the results calculated by IQ-TREE, the model for the rDNA partition was set as ‘GTR’, and the models for most amino acid partitions were set as ‘LG’ (see details in Data S3). Bootstrap values were calculated by BOOSTER [19] with default settings.

### 2.3. Reconstruction of Ancestral Sequences of rDNAs

The gene sets of 16S, 5S, and 23S rRNAs were manually optimized according to the corresponding secondary structures, which were downloaded from the Comparative RNA Web Site and Project (https://crw-site.chemistry.gatech.edu/, accessed on 5 April 2021). Then the four types of bases and gaps were changed into number 0 to 4. The ancestral sequences of 16S, 5S, and 23S rRNAs of archaea, bacteria, and LUCA, respectively, were reconstructed by Mesquite using the likelihood method. For each site, the base with the highest likelihood value was employed. The numbers were changed back to ‘A’, ‘U’, ‘G’, ‘C’, and ‘N’, while ‘N’ referred to the gap.

### 2.4. Searching and Filtering the Short Fragments

In the concatenated 16S, 5S, and 23S rRNA sequences of LUCA, the ‘A’ and ‘G’ were converted to ‘R’, the ‘U’ and ‘C’ were converted to ‘Y’. Fragments (in the AUGC level or RY level) with same length and sequence (one type) were searched along the concatenated sequence. All potential types were thoroughly searched. Different lengths were taken into consideration. This was done with a custom script (Data S4) written in Python3 (https://www.python.org/, accessed on 1 July 2019). In other words, all *k*-mers (in AUGC level or RY level) were successively searched along the concatenated sequence, and the *k*-mers with unique sequences or purine–pyrimidine arrangements were filtered, while the same two or more *k*-mers were selected. In the RY level and the AUGC level, short fragments of lengths from 2 to 16 and 2 to 13 were searched, respectively. When the length was 16 in the RY level or 13 in the AUGC level, none of the short fragments with the same length and sequence was found. The concatenated rRNA sequences of archaeal and bacterial ancestors were carried out with the same method as aforementioned, respectively. The short fragments with different lengths were also searched. The short fragments shared by all of the three treatments were kept due to some adjacent short fragments that overlapped with each other. If the overlaps were higher than 40%, only one of them was kept; otherwise, both of the two adjacent short fragments were retained.

Moreover, the 16S, 5S, and 23S rRNAs of 7 extant species including *Pyrococcus abyssi*, *Escherichia coli*, *Saccharomyces cerevisiae*, *Phalansterium solitarium*, *Drosophila melanogaster*, *Arabidopsis thaliana*, and *Homo sapiens* were aligned with the LUCA sequences and those of archaea and bacteria, respectively. The orthologous regions corresponding to the short fragments mentioned above were recognized.

### 2.5. Mapping Short Fragments on Secondary and Tertiary Structures

In order to investigate the characters and spatial positions of short fragments in a visualized method, they were mapped on secondary and tertiary structures of rRNAs. The secondary structures of 16S, 5S, and 23S rRNAs of *E. coli* were downloaded from the Comparative RNA Web Site and Project (https://crw-site.chemistry.gatech.edu/, accessed on 5 April 2021). The bases in secondary structures of *E. coli* were replaced by the orthologous base of LUCA in Adobe Illustrator (https://www.adobe.com/products/catalog.html, accessed on 13 June 2018). The short fragments were then mapped on the secondary structures with colored curves in Adobe Illustrator. The tertiary structures of *E. coli* were downloaded from the Protein Data Bank (PDB ID: 7N1P) (accessed on 12 October 2021) [20]. The short fragments were mapped on the tertiary structures with colored lines.

## 3. Results

### 3.1. Phylogenetic Relationships of Archaea and Bacteria

**Figure 2:**
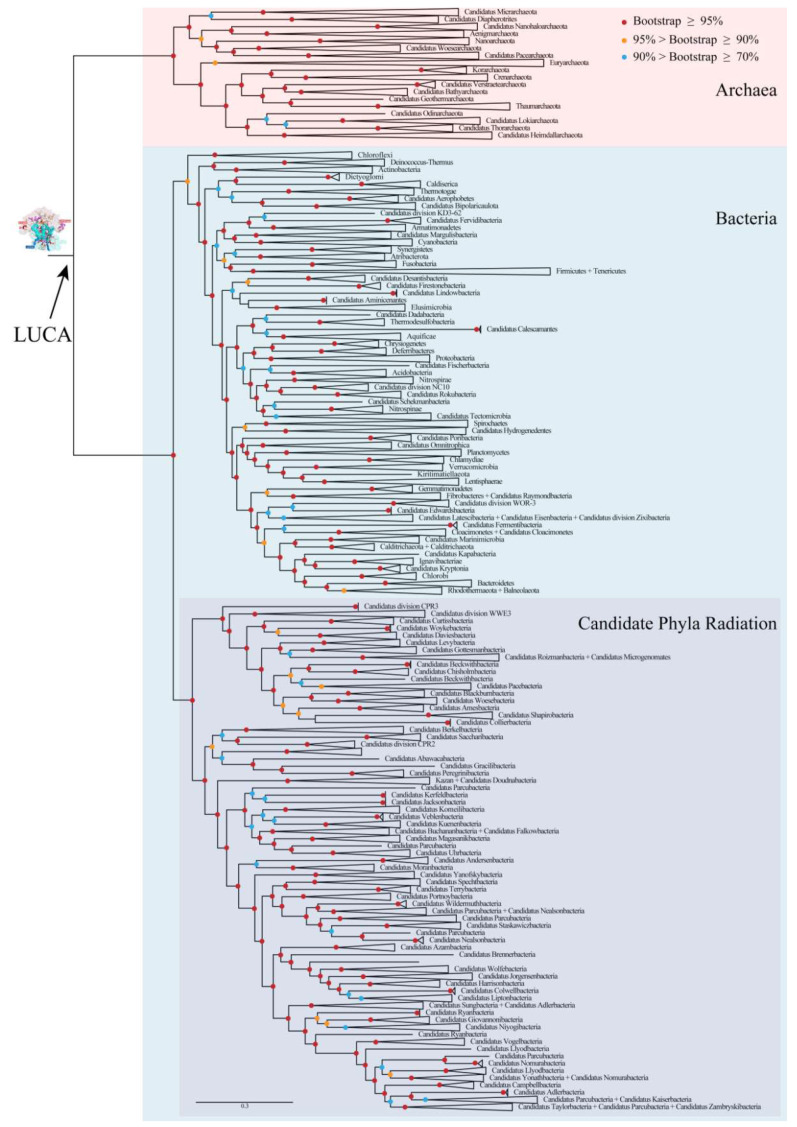
Phylogenetic relationships of archaea and bacteria. Branches were grouped to display the tree at the phylum level. Bootstrap values for this ML(Maximum Likelihood) tree were indicated by colored circles on nodes. LUCA: last universal common ancestor. The ML-tree with all branches is shown in Figure S1.

To explore the characters of LUCA rRNAs, phylogenetic relationships of archaea and bacteria were reconstructed based on a gene set containing 166 genes of 531 species. These species belonged to 153 known phyla and candidate phyla, which covered nearly all known phyla and candidate phyla of archaea and bacteria after taking different taxonomic systems into consideration [21,22] (Table S1). No eukaryotes were sampled due to the well-accepted theory that its phylogenetic position is nested in archaea, with a close relationship to Asgard [23,24]. The concatenated matrix, which was used to reconstruct the phylogenetic relationships consisted of 34,417 positions, including 31,143 aligned amino acid positions and 3274 aligned nucleotide positions. The COG (cluster of orthologous group) name for each gene and the statistical information for the concatenated matrix for each species are shown in Table S2. The overall coverages of the 3 rRNA genes and the 163 protein coding genes were 74.14% and 66.73%, respectively, and those of the nucleotide sites and the amino acid sites were 73.43% and 64.39%, respectively. A well-resolved tree with bootstrap values for most nodes higher than 90% was obtained (Figure 2 and Figure S1). The ML-tree with all branches is shown in Figure S1. Archaea, the candidate phyla radiation (CPR) of bacteria, non-CPR bacteria, and almost all phyla were found to be monophyletic with bootstrap values of 100%. The reliable phylogenies of archaea and bacteria provided a sound base for the following reconstruction of ancestral rRNA sequences of the LUCA and, thereafter, the analysis of self-similarity.

### 3.2. Ancestral Sequences of 16S, 5S, and 23S rRNAs

Based on the well-resolved phylogenetic relationships, the sequences of 16S, 5S, and 23S rRNAs of the archaeal ancestor, bacterial ancestor, and LUCA were reconstructed utilizing the likelihood method (Figure S2; Table S3). The lengths of 16S rRNA, 5S rRNA, and 23S rRNA of LUCA were 1472, 120, and 2836 nucleotides, respectively.

### 3.3. Short Fragments Shared by 16S, 5S, and 23S rRNAs

The self-similarity of ancestral rRNA sequences were analyzed to search for the repeat units with the same purine–pyrimidine arrangements. In other words, *k*-mers were compared with each other, and those with the same purine–pyrimidine arrangements were searched. Short fragments of lengths from 2 to 16 were searched.

In the cases in which the length was 16, no short fragments with similarity existed. With the length of 15, two short fragments with the arrangement of ‘RRRRRYYRRYRRRRR’ at the 887–901 sites of 16S rRNA and the 41–55 sites of 23S rRNA were reached (Table S4). Thus, when the length was 15, a set of two short fragments with no overlap was obtained, which belonged to 1 type of purine–pyrimidine arrangement. In the cases in which lengths ranged from 2 to 14, at least one short fragment could be found either in 16S, or 5S, or 23S rRNAs (Table S4), and some short fragments belonging to different types overlapped with others. Moreover, it was inevitable that when the length was 14, two short fragments with the arrangement of ‘RRRRRYYRRYRRRR’ at the 887–900 sites of 16S rRNA and the 41–54 sites of 23S rRNA would be reached, while another two short fragments with the arrangement of ‘RRRRYYRRYRRRRR’ at the 888–901 sites of 16S rRNA and the 42–55 sites of 23S rRNA would be reached (Table S4). Each one of the two fragment types (RRRRRYYRRYRRRR and RRRRYYRRYRRRRR) had two overlaps of 13 nucleotides in length, one ranging from 888 to 900, and the other ranging from 42 to 54. These two kinds of overlaps were seen as repeat information, which should be filtered, and the terms' positive and pseudo-positive results were employed. Positive results were the fragments with no or slight overlaps, which may have participated in the formation of the LUCA ribosome. In contrast, the pseudo-positive results were those heavily overlapped with others and did not participate in the formation of the LUCA ribosome.

**Figure 3:**
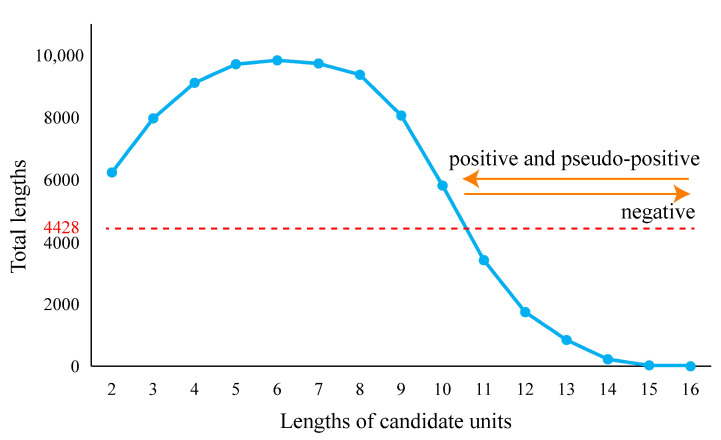
The total lengths of candidate units. The total length is equal to the length of the candidate unit multiplied by the number of candidate units. The total length of ancestral 16S, 5S, and 23S rRNA sequences of LUCA was 4428 nucleotides (marked by red line).

Pseudo-positive results can be extraordinarily heavy in some sets of fragments. When the fragment length belonged to the section from 2 to 10, the total length of each fragment set (length of fragment multiplied by the number of fragments) was longer than 4428 nucleotides, which was the total combined length of 16S, 5S, and 23S rRNAs of LUCA (Figure 3). When the lengths increased from 11 to 15, fewer pseudo-positive and fewer positive results were obtained. Moreover, eleven was the longest length with the short fragment set that covered all types of the known functional sites. Simultaneously considering the relative length of a fragment set to 4428, the balance between the positive and pseudo-positive results, and the most concise manner, eleven appeared to be the most appropriate representative length to display the characters of LUCA rRNAs.

**Figure 4:**
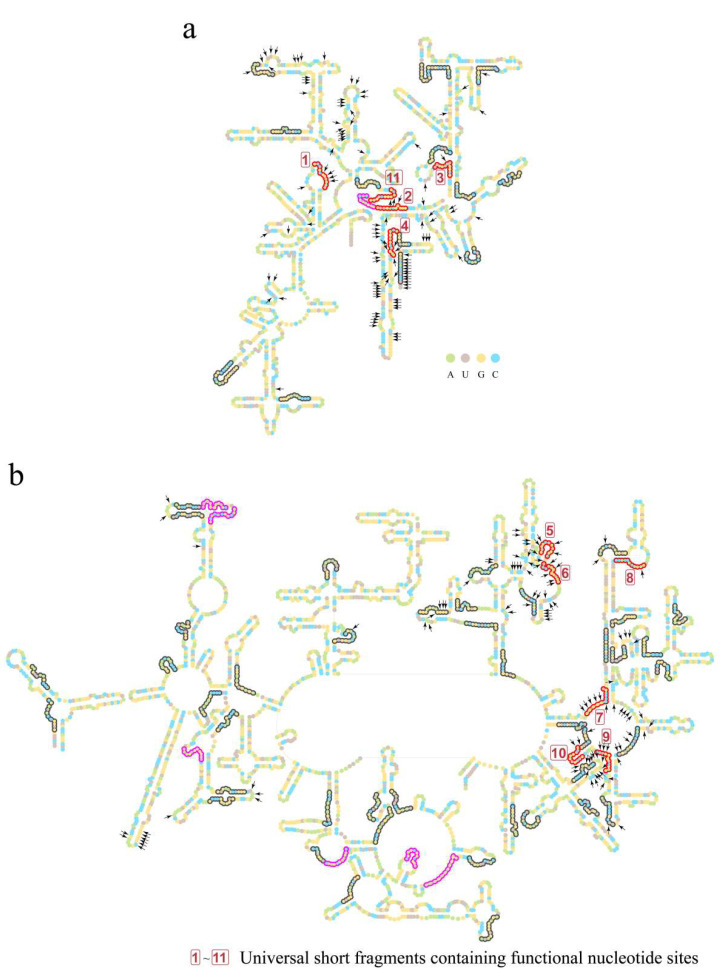
Short fragments with 11 nucleotides in the RY level on secondary structures of LUCA rRNAs. (**a**) Secondary structure of 16S rRNA of LUCA. (**b**) Secondary structures of 5S rRNA (left) and 23S rRNA (right) of LUCA. Bases are indicated by colored circles. The black arrows mark the 226 nucleotides that were orthologous to the nucleotides performing functions in the ribosome (in total, 230 functional nucleotides were summarized, while 4 nucleotides’ orthologous sites were gaps in the reconstructed LUCA rRNA chains. Thus, a total of 226 nucleotides was marked). Short fragments are outlined by colored curves. Red and magenta curves outline the 18 universal short fragments, which were conserved across at least 5 of archaea, bacteria, protists, fungi, plants, and animals. Red curves and boxed numbers (from 1 to 11) outline and mark the 11 universal short fragments whose orthologous sequences of *E. coli* contain nucleotides performing functions in the ribosome. Magenta curves outline the other 7 universal short fragments containing no functional sites. An additional 57 short fragments are outlined by gray curves.

With the length of 11, 75 short fragments in total belonging to 35 types of purine–pyrimidine arrangements were obtained after filtering at the threshold of 40% overlaps (Table 1). Twenty-one short fragments belonged to the ancestral 16S rRNA, two belonged to the ancestral 5S rRNA, and fifty-two belonged to the ancestral 23S rRNA (Figure 4 and Figures S3–S5; Table S5).

### 3.4. Short Fragments Largely Covered the PTC and the Inter-Subunit Interface of Ribosome

**Figure 5:**
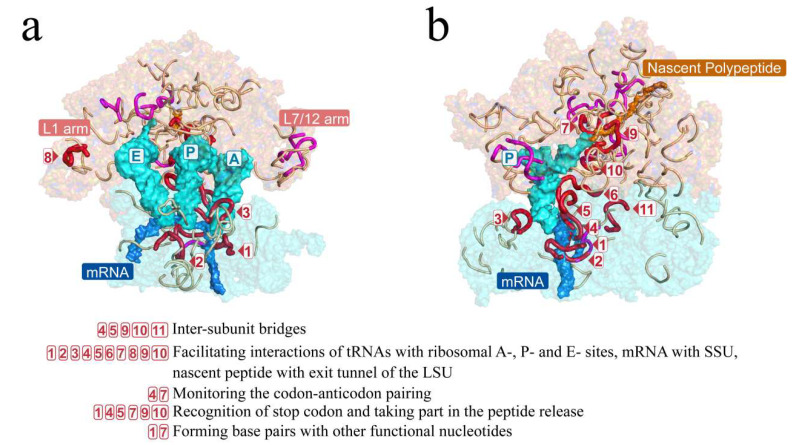
Short fragments with 11 nucleotides in the RY level largely covered the key functional areas of the ribosome. The ribosomal tertiary structure of *E. coli* was mapped with the short fragments and is displayed on the front (**a**) and side (**b**) views. Short fragments are shown by colored lines with red, magenta, and gray. The short fragments marked in three colors and boxed numbers correspond to those in Figure 4. Red and magenta mark the 18 universal short fragments that were conserved across at least 5 of archaea, bacteria, protists, fungi, plants, and animals. Red and boxed numbers mark the 11 universal short fragments whose orthologous sequences of *E. coli* contain functional nucleotide sites. The functions of nucleotides contained in the 11 fragments are listed below the tertiary structures. Magenta marks the other 7 universal short fragments containing no functional site. An additional 57 short fragments are marked in gray. The L1 and L7/12 arms were functionally important domains in the ribosome.

Short fragments were mapped to the tertiary structures of rRNAs of the *E. coli* ribosome (Figure 5). The short fragments clustered around and largely covered the inter-subunit interface, the PTC, and the nascent peptide tunnel, which constitute the functional center of the ribosome. Such a coincident pattern in the tertiary structures illustrated that these short fragments may be crucial in the LUCA ribosome.

### 3.5. The Conservativeness of Short Fragments

The positions and purine–pyrimidine arrangements of nearly half (36/75) of the short fragments were the same to their orthologous sequences of *P. abyssi* (Archaea) and *E. coli* (Bacteria) rRNAs. They were conserved in the extant species of archaea and bacteria. Surprisingly, 18 of them were highly conserved across archaea, bacteria, protists, fungi, plants, and animals, or at least five of the six kingdoms (Table S5). These 18 were referred as the 18 universal or conserved short fragments in the following descriptions.

The conservativeness of the 36 short fragments suggested that they may perform crucial functions in the catalytic process of the ribosome, particularly the 18 highly conserved short fragments that contained three short fragments marked with boxed 7, 9, and 10 formed parts of the peptidyl transferase center (PTC) (Figure 4, Figure 5 and Figure S5).

### 3.6. Functional Attributes of the Short Fragments Checked with the Knowledge of Structural Biology

**Figure 6:**
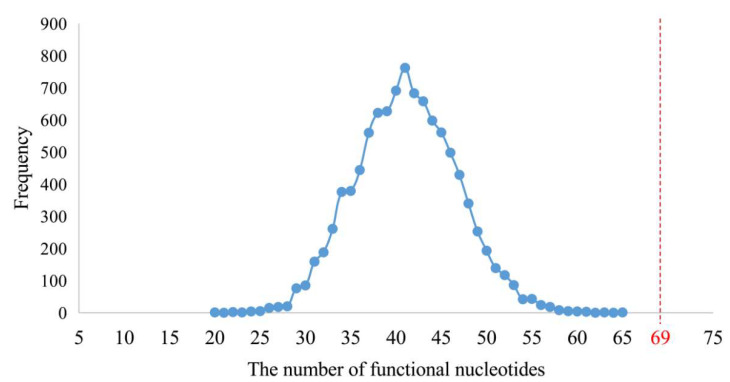
Number of randomly sampled functional nucleotide sites from the three rRNAs. The numbers of functional nucleotide sites contained in the randomly sampled 805 nucleotide sites are shown in blue. In total, 230 functional nucleotide sites of contemporary rRNAs were summed up. Sixty-nine of them were located in the 75 short fragments (marked in red).

In order to test the functional attributes of these 75 short fragments, their orthologous sequences of *E. coli* were used for mapping. At the same time, the functional nucleotides and sites of contemporary rRNAs were summarized from the structural and functional studies of the *E. coli* and other species’ ribosomes (Figure 4 and Figures S3–S5; Table S6). In total, 230 functional sites of nucleotides were summed up. Sixty-nine of them were located in the 75 short fragments (Table S6), whose total length was 805 nucleotides. Although 69 out of 230 functional sites did not appear via effective data mining, such a result was not likely to be reached by random sampling (Figure 6).

Moreover, 34 of the 69 functional nucleotide sites were located in the 18 universal short fragments. In fact, 11 among the 18 universal short fragments contained the 34 functional nucleotide sites (these 11 short fragments are marked in red or boxed numbers in Figure 4, Figure 5 and Figure S2), while the other 7 universal short fragments contained no functional nucleotide sites known up to now (these 7 short fragments are marked in magenta in Figure 4, Figure 5 and Figure S2). The functions performed by the 34 nucleotide sites include inter-subunit bridges, facilitating interactions of tRNAs with ribosomal A-, P-, and E- sites, facilitating interactions of mRNA with SSU, facilitating interactions of nascent peptide with the exit tunnel of the LSU, monitoring the codon–anticodon pairing, recognition of stop codon, as well as forming base pairs with other functional nucleotides (Figure 5; Table 2 and Table S6). Such inclusions of short fragments with all types of functional nucleotide sites except those interacting with GTPase factors confirmed that the short fragments were likely to take part in the formation of the LUCA ribosome.

In the statistical test, a total of 805 positions of nucleotides was randomly sampled from the complete sequences of the three rRNAs of *E. coli* with 4566 nucleotides by a module written in Python3 (https://www.python.org/, accessed on 1 July 2019) (Data S5). The 805 randomly sampled positions were compared to those of the 230 functional nucleotides, which were summarized within the structural and functional studies of extent species ribosome as described above. Then the number of sampled functional nucleotides’ positions was counted. The random sampling and counting were repeated 10,000 times. The average number of sampled functional nucleotides’ positions was 41, which was far less than 69 (Figure 6). It proved that 69 out of 230 functional nucleotides represented effective data mining.

### 3.7. Alternative Strategy of AUGC Level

Short fragments in the AUGC level were also searched within the 16S, 5S, and 23S rRNA sequences (Table S7). Short fragments of lengths from 2 to 13 were searched, respectively. When the length was 13, no short fragments existed. In cases in which the length ranged from 7 to 12, no short fragments could be found in 5S rRNA. In addition, when the length ranged from two to six, at least one short fragment could be found either in 16S, 5S, or 23S rRNAs (Table S7). Overlaps and pseudo-positive results also existed in the strategy of the AUGC level, especially in the short fragment sets searching with the lengths from two to five, whose total lengths were longer than the total combined length of three rRNAs of LUCA (4428 nucleotides). Moreover, six was the longest length, with the short fragment set covering nearly all types of the known functional sites and simultaneously located in the 16S, 5S, and 23S rRNAs. Considering the relative length of a fragment set to 4428, the balance between the positive and pseudo-positive results and the most concise manner, six appeared to be the most appropriate representative length in the AUGC level.

With the length of six, a total of 136 short fragments were reached after searching and filtering at the threshold of 40% overlaps (Table 1). Thirty-four short fragments belonged to the ancestral 16S rRNA, two belonged to the ancestral 5S rRNA, and one-hundred belonged to the ancestral 23S rRNA (Figure S6; Table S8). Among the 136 short fragments, the sequences of 47 short fragments were conserved in the extant species of archaea and bacteria, while 29 of them were highly conserved across at least five kingdoms (Table S8).

In the functional attributes testing, forty-eight of the total summarized functional sites were located in the 136 short fragments. Among 136 of them, the 29 conserved short fragments contained 12 short fragments whose orthologous sequences of *E. coli* contained functional nucleotides which took part in all the known activities of the ribosome except one, which takes part in the peptide release (marked in red in Figure S6 and Table S9). The other 17 conserved short fragments contained no functional site known up to now (marked in magenta in Figure S6 and Table S9).

Comparing the former set of short fragments with 11 nucleotides in the RY level and the latter set of short fragments with 6 nucleotides in AUGC level, conformance of the two sets was considerable such that about two-thirds of the total short fragments (56–68%) or universal short fragments (66–67%) were overlapped or adjacent (Table 1; Figure S7). The sets of short fragments in the AUGC level showed similar distribution and function patterns, namely that short fragments or local similarities were shared by 16S, 5S, and 23S rRNAs, and they were conserved and contained nucleotide sites performing all types of ribosomal functions except one.

## 4. Discussion

The ribosome has been regarded as a molecular living fossil that gives us a glimpse into the chemical origins of life [10]. The initial primitive ribosome was probably composed entirely of RNAs [59], while in extant cellular organisms, the ribosome RNAs were quite complex with different lengths and mixed with proteins. Thus, the origins of rRNAs are difficult to envision.

To investigate the origins of ribosomal RNAs in the approaches of phylogenetics and ancestral state reconstruction, the phylogenetic relationships of archaea and bacteria were reconstructed based on 531 species covering almost all known phyla and candidate phyla. The relationships of archaeal superphyla, the candidate phyla radiation (CPR) of bacteria and non-CPR bacteria were consistent with the previous studies [13,60,61]. Based on the phylogeny of archaea and bacteria with bootstrap values of most nodes higher than 90%, full lengths of 16S, 5S, and 23S rRNA sequences of LUCA were reconstructed for the first time.

Within the concatenated sequence of the three ancestral rRNAs in the RY level, the set containing 75 short fragments with a length of 11 gave the most appropriate and concise representative (as mentioned in the introduction). These short fragments were shared by the 16S, 5S, and 23S rRNAs of LUCA. Thirty-six of the 75 short fragments were conserved across archaea and bacteria, and 18 of the 36 were highly conserved across at least 5 kingdoms (Table 1). Consider that any one of the transversion, insertion, deletion, or other changes during the 4 billion years’ evolution process would erase the conserved nature of 36 short fragments, as they may have been vitally important during the evolution of rRNAs. The functional attributes of short fragments were tested, and it was verified that the 75 short fragments or even 18 of them covered all types of functional nucleotide sites of the ribosome except one. These results showed that they may take part in the origin and evolution of the LUCA ribosome.

Another strategy to search for repeat short fragments in the AUGC level was performed, and similar results reappeared. On the one hand, some different short fragments at different positions were found within the ancestral sequences in the RY and AUGC levels. Compared to the AUGC level, searching for short fragments in the RY level was more inclusive, and thus more positive results could be reached. At the same time, pseudo-positive results could increase as by products (Table 3). By contrast, searching for short fragments in the AUGC level was stricter, and thus fewer positive results could be reached (Table 3). On the other hand, although differences existed in the two strategies, the distribution pattern and function attributes of short fragment sets were quite similar in the RY and AUGC levels.

Except for the searching strategy, length was another factor which could affect the number and positions of short fragments. Fragment sets searched with longer lengths contained fewer pseudo-positive results, and fewer positive results were obtained at the same time (Table 3). On the contrary, fragment sets searched with shorter lengths contained more positive and pseudo-positive results, which skewed the results toward redundant pseudo-positives. No matter which length was deployed to search for short fragments, positive and pseudo-positive results simultaneously existed, and local similarities shared by the three rRNAs could be reappeared by them, just with different portions. The set of longest short fragments which simultaneously distributed on the three rRNAs and covered nearly all types of functional nucleotide sites was the most appropriate set to exhibit the positional and functional characters in the most concise manner, just like the two sets of 75 short fragments with 11 nucleotides in the RY level, and 136 short fragments with 6 nucleotides in the AUGC level.

The short fragments obtained from the LUCA rRNAs may be relics of the RNA world because the ribosomes of extant species have inherited the most important parts of the mechanism of that initial function [62], and thus the corresponding structures or sequence parts remained in the LUCA ribosome. As for the formation of short fragments or self-similarities among 16S, 5S, and 23S rRNAs, two possible pathways existed, i.e., convergence vs. inheritance. To examine the possibility of convergence, functional nucleotide sites contained in two or more short fragments belonging to each same type were reviewed, and they corresponded to different functions or no function. In other words, in each type of two or more short fragments, no functional nucleotide site performed absolutely the same functions. For example, short fragments ‘CAGUUC’ (code number 1-1283 corresponding to the 16S rRNA sequence of *E. coli* at sites 1298–1303) and ‘CAGUUC’ (code number 2-2797 corresponding to the 23S rRNA sequence of *E. coli* at sites 2601–2606) belonged to the type ‘CAGUUC’ (Table S8). The former one contained a nucleotide corresponding to the 1300 site of *E. coli* 16S rRNA, which takes part in the interaction with mRNA. The latter one contained two nucleotides corresponding to the 2602 and 2603 sites of *E. coli* 23S rRNA, which take part in the interaction with tRNA, peptide release, and inter-subunit bridges (Table S9). No selection pressure of convergence existed between the short fragments with the same sequence but different positions. If the short fragments belonging to a same type formed by random convergence (coincidence), such a hypothesis is up against far more complex processes with accumulated mutations. Hence, short fragments with the same sequences or arrangements seem difficult to have formed through convergence.

Another possible pathway of inheritance seems more concise and thus convincing. In the RNA world, the self-replicated fragments could have been preserved in their descendants [3] and thus increased in number. All fragments may have floated in the original soup. The fragments with larger proportion would have been more likely to take part in or contribute to the formation of rRNA chains. The local similarities shared by the three rRNAs provided new evidence for the existence of self-replicating systems [1].

Although our results and assumptions were different from the previous studies exploring the origins and evolution of 16S and/or 23S rRNAs, they were not contradictive of the known hypotheses. Previously, the hierarchical theory [9], the discussion of the protoribosome concept [63,64,65], and the stepwise accretion theory [10,66,67] have provided insights into the aspects of A-minor interaction, conservativeness level, and insertion fingerprints, respectively. These theories have provided a pattern that the PTC was an ancient core and different units were incorporated in the core over time to form the final rRNAs. In comparison, our methods of phylogenetics and ancestral state reconstruction, our results of local self-similarities shared by the 16S, 5S, and 23S rRNAs of LUCA, and our testing by function nucleotide sites in extant species were quite different from the previous ones. Moreover, the universal short fragments with function attributes reached here covered the inter-subunit interface, the nascent peptide tunnel, and the PTC, which was regarded as the ancient core of ribosome. Some short fragments may act as incorporated units or parts of them to take part in the formation of rRNA chains.

## 5. Conclusions

It is considered that the ribosome originated in the RNA world [8], fully matured at LUCA [8,10], and diversified and appeared tangible in extant species. Here we reconstructed the 16S, 5S, and 23S rRNA sequences of LUCA, pinpointed the short fragments with the same sequences, and demonstrated the local similarities shared by the three rRNAs for the first time based on phylogenetics, ancestral state reconstruction, data mining of self-similarities, confirmation of the short fragments’ conservativeness, and checking function attributes of short fragments. The results indicated that short fragments may act as elements in the formation of LUCA rRNAs. A bold conjecture was proposed that the three rRNAs might originate in a similar pathway in which the short fragments with different lengths acted as RNA units or parts of them. These RNA units constituted the PTC core and then the rRNA chains. In the future, the possibility of this conjecture can be practically tested by a simulating experiment.

## Figures and Tables

**Table 1:** Overview of the lengths, positions, conservativeness, and function attributes of short fragments in RY and AUGC levels.

	RY	AUGC
Length range of short fragment	2–15	2–12
Representative length in the most concise manner	11	6
Total number of short fragments with the representative length	75	136
Percentage of the overlapped or adjacent short fragments	68%	56%
Number of short fragments that belonged to the 16S rRNA	21	34
Number of short fragments that belonged to the 5S rRNA	2	2
Number of short fragments that belonged to the 23S rRNA	52	100
Number of short fragments conserved across archaea and bacteria	36	47
Number of universal short fragments (conserved across 5 or 6 kingdoms)	18	29
Percentage of the overlapped or adjacent universal short fragments	67%	66%
Number of functional sites located in the total short fragments	69	48
Number of universal short fragments that contained functional sites	11	12
Number of universal short fragments that contained no functional sites	7	17

**Table 2:** Functions of nucleotide sites in short fragments.

Function	N-Box	N-F75	References
Interaction with tRNA in A-, P-, and E-sites	1, 2, 3, 4, 5, 6, 8, 9, 10	529 (1-491), 530 (1-491), 531 (1-491), 532 (1-491), 2583 (2-2772), 2602 (2-2788), 1492 (1-1478), 1493 (1-1478), 1494 (1-1478), 1916 (2-2101), 1918 (2-2101), 1926 (2-2114), 2585 (2-2772), 926 (1-891), 956 (1-924), 2584 (2-2772), 2602 (2-2788), 2169 (2-2359), 1913 (2-2101), 2609 (2-2796), 2506 (2-2692), 2555 (2-2750), 2501 (2-2692), 2603 (2-2796), 2505 (2-2692), 693 (1-654), 2116 (2-2304)	[25,26,27,28,29,30,31,32,33]
Interaction with mRNA	1	532 (1-491), 693 (1-654), 1156 (1-1132), 1533 (1-1516), 1532 (1-1516), 1530 (1-1516), 1534 (1-1516), 1535 (1-1516), 1536 (1-1516), 1537 (1-1516), 1538 (1-1516), 1539 (1-1516), 1540 (1-1516)	[27,34,35,36,37]
Interaction with mRNA–tRNA minihelix	1, 4	1492 (1-1478), 1493 (1-1478), 530 (1-491)	[38]
Interaction with nascent peptide	7, 9	2062 (2-2249), 2585 (2-2772), 2506 (2-2692), 2609 (2-2796), 1614 (2-1797)	[39,40]
Monitoring the codon–anticodon pairing and maintaining translational fidelity	4	1492 (1-1478), 1493 (1-1478)	[41,42,43,44,45,46]
Co-translational monitoring of nascent peptide chains inside the exit tunnel	7	2062 (2-2249)	[43]
Recognition of stop codon	1, 4, 5	1913 (2-2101), 530 (1-491), 1493 (1-1478)	[47]
Nascent peptide tunnel	7	2058 (2-2249), 2059 (2-2249), 2060 (2-2249), 2061 (2-2249), 2062 (2-2249), 2063 (2-2249)	[30,48]
Taking part in the peptide release	9, 10	2585 (2-2772), 2602 (2-2788)	[30,49]
Inter-subunit bridges	4, 5, 9,10, 11	900(1-870), 901(1-870), 1493(1-1478), 1495(1-1478), 1496(1-1478), 1702(2-1884), 1703(2-1884), 1704(2-1884), 1705(2-1884), 1912(2-2101), 1913(2-2101), 1923(2-2114), 1928(2-2114), 1929(2-2114), 1932(2-2114), 1933(2-2114), 1960(2-2148), 1961(2-2148), 1962(2-2148), 2506(2-2692), 2585(2-2772), 2602(2-2788)	[50,51]
Forming base pairs with other functional nucleotides	1, 7	2061 (2-2249), 2063 (2-2249), 530 (1-491), 2499 (2-2692)	[25,30,52]
Interacting with GTPase factors (EF-G, EF-Tu, IF2, RF3)		2653 (2-2845), 2654 (2-2845), 2655 (2-2845), 2656 (2-2845), 2657 (2-2845), 2658 (2-2845)	[53,54,55,56,57,58]

N-Box: Boxed number corresponding to that in Figure 5. N-F75: Functional nucleotide sites contained in the 75 short fragments. The part outside the bracket refers to the functional nucleotide set in the rRNAs of *E. coli*. The part inside the bracket is the code number of the corresponding short fragment’s first nucleotide. The number before ‘-’ is the location, where ‘1’ is for 16S rRNA and ‘2’ is for 23S rRNA. The number after ‘-’ refers to the number of ancestral sequences with gaps.

**Table 3:** Comparisons of two strategies and different lengths.

	Fragment Set with the Same Length		Fragment Set in the Same Level	
	RY Level	AUGC Level	Short	Long
Positive results	More	Less	More	Less
Pseudo-positive results	More	Less	More	Less
Overlaps	More	Less	More	Less
Functional sets	More	Less	More	Less

## Data Availability

The data presented in this study are openly available in DataOpen at http://dataopen.info/home/datafile/index/id/254 accessed on 3 May 2022 and the Supplementary Materials.

## Supplementary Materials

The following supporting information can be downloaded at: https://www.mdpi.com/article/10.3390/biology11060837/s1, Figure S1: Phylogenetic relationships of archaea and bacteria. The archaea and bacteria were rooted with each other: (a) the general topology of the tree; (b) the topology of archaea; (c) the topology of non-CPR bacteria; (d) the topology of CPR. Figure S2: Ancestral 16S, 5S, and 23S rRNA sequences of LUCA. The sequences of 16S rRNA (a), 5S rRNA (b), and 23S rRNA (c) of LUCA. The first number in each line is the site of the first nucleotide. Short fragments which were conserved across at least 5 of archaea, bacteria, protists, fungi, plants, and animals are marked in red and magenta. Red is for the 11 short fragments whose orthologous sequences of *E. coli* contain functional nucleotide sites. Magenta is for the other 7 short fragments containing no functional nucleotide sites. The other 57 short fragments are marked in gray. Figure S3: Secondary structure of 16S rRNA of LUCA. Parts of Figure 4. Figure S4: Secondary structures of 5S rRNA and 5′-half of 23S rRNA. Parts of Figure 4. Figure S5: Secondary structure of 3′-half of 23S rRNA. Parts of Figure 4. Figure S6: Short fragments with the length of 6 in AUGC level on secondary structures of LUCA rRNAs. (a) Secondary structure of 16S rRNA of LUCA. (b) Secondary structures of 5S rRNA (left) and 23S rRNA (right) of LUCA. Red and magenta curves outline the universal short fragments which were conserved across at least 5 of archaea, bacteria, protists, fungi, plants, and animals. Red curves outline the universal short fragments containing functional nucleotide sites. Magenta curves outline the other universal short fragments containing no functional nucleotide sites. The other short fragments are outlined by gray curves. Figure S7: Comparison of the two sets which contain 75 short fragments with 11 nucleotides in the RY level (marked by red curves), and 136 short fragments with 6 nucleotides in the AUGC level (marked in black circles). Supplementary Table S1: Information of the sampled species. Supplementary Table S2: The detailed information for each orthologous gene. The COG number for each protein, the presence/absence for each rDNA and protein, together with the overall number of sites for each species are shown. The sheet named ‘3 rRNA genes’ contains the statistics about the 16S, 5S, and 23S rRNA genes. Another sheet named ‘163 PCGs’ contains the statistics about the 163 protein coding genes. The 130 proteins coded as COG number were the ones searched and filtered in this study. The other 33 proteins coded as pXXXX were obtained from a previous study, which were justified informative in the phylogenetic analyses of bacteria and archaea [13]. In each of the two sheets, the parts inside the bracket are the numbers of Ns and indels, and the part outside the bracket is the total positions. The ‘/’ indicates the absence of the corresponding genes. Supplementary Table S3: The 16S, 5S, and 23S rRNA sequences of the ancestor of archaea, ancestor of bacteria, LUCA, and 7 extant species. Nucleotides in the same column are aligned to be orthologous. The code number of the three rRNA nucleotides contain two parts. The number before ‘-’ is the location, where ‘1’ is for 16S rRNA, ‘5’ is for 5S rRNA, and ‘2’ is for 23S rRNA. The number after ‘-’ refers to the number of ancestral sequences with gaps. Supplementary Table S4: The short fragment sets (purine–pyrimidine level) before filtering are listed in sheets that are named as the length of each short fragment. The positions and purine–pyrimidine arrangements are included. Short fragments: The purine–pyrimidine arrangements of short fragments. N-Sf: The code number of the first nucleotide of the short fragment. The number before ‘-’ is the location where ‘1’ is for 16S rRNA, ‘5’ is for 5S rRNA, and ‘2’ is for 23S rRNA. The number after ‘-’ refers to the number of ancestral sequences with gaps. Species: The purine–pyrimidine arrangements of short fragments are the same as the orthologous sequences of the listed species. N-sp: The number of listed species. Supplementary Table S5. Short fragments with the length of 11 in the RY level. Short fragments were searched from the 16S, 23S, and 5S rRNA sequences of LUCA, which were converted from ‘A’, ‘G’ to ‘R’ and converted from ‘U’, ‘C’ to ‘Y’. N-Sf: The code number of the first nucleotide of the short fragment. The number before ‘-’ is the location where 1’ is for 16S rRNA and ‘2’ is for 23S rRNA. The number after ‘-’ refers to the number of ancestral sequences with gaps. N-Ec: The number of the orthologous nucleotide of the short fragment’s first nucleotide in the rRNAs of *E. coli*. Species: The purine–pyrimidine arrangements of short fragments are the same as the orthologous sequences of the listed species. N-sp: The number of listed species. The short fragments with red, magenta, and black fonts correspond to those marked by red, magenta, and gray, respectively, in Figure 4 and Figure 5. Supplementary Table S6: Functions of nucleotides in short fragments (RY level and 11 in length). N-Box: Boxed number corresponding to that in Figure 5. N-F18: Functional nucleotide sites contained in the 18 short fragments. N-F57: Functional nucleotide sites contained in the other 57 short fragments. N-F18 and N-F57: The part outside the bracket is the number in the 16S and 23S rRNAs of *E. coli*. The part inside the bracket is the code number of the corresponding short fragment’s first nucleotide. The number before ‘-’ is the location where ‘1’ is for 16S rRNA and ‘2’is for 23S rRNA. The number after ‘-’ refers to the number of ancestral sequences with gaps. N-NF75: Positions of functional nucleotide sites not contained in the 75 short fragments. Supplementary Table S7: The short fragment sets (four nucleotide levels) before filtering are listed in sheets that were named as the length of each short fragment. The positions and sequences are included. Short fragments: the sequences of short fragments. N-Sf: The code number of the first nucleotide of the short fragment. The number before ‘-’ is the location where ‘1’ is for 16S rRNA, ‘5’ is for 5 rRNA, and ‘2’ is for 23S rRNA. The number after ‘-’ refers to the number of ancestral sequences with gaps. Species: The sequences of short fragments are the same as the orthologous sequences of the listed species. N-sp: The number of listed species. Supplementary Table S8: Short fragments with the length of 6 in the AUGC level. Short fragments were searched from the 16S, 23S, and 5S rRNA sequences of LUCA at the AUGC level. N-Sf: The code number of the first nucleotide of the short fragment. The number before ‘-’ is the location where ‘1’ is for 16S rRNA and ‘2’ is for 23S rRNA. The number after ‘-’ refers to the number of ancestral sequences with gaps. N-Ec: The number of orthologous nucleotides of the short fragment’s first nucleotide in the rRNAs of *E. coli.* Species: The sequences of short fragments are the same as the orthologous sequences of the listed species. N-sp: The number of listed species. The short fragments with red, magenta, and black fonts correspond to the ones marked by red, magenta, and gray, respectively, in Figure S6. Supplementary Table S9: Functions of nucleotides in short fragments (AUGC level and 6 in length). N-F136: Functional nucleotide sites contained in the 136 short fragments. The parts outside the bracket refer to the functional nucleotide site in the 16S and 23S rRNAs of *E. coli*. The part inside the bracket is the code number of the corresponding short fragment’s first nucleotide. The bold code number marks the short fragments which were conserved across 5 or 6 kingdoms.

## References

[1] Gilbert W. (1986). Origin of Life: The RNA World. Nature.

[2] Yarus M. (2011). Getting Past the RNA World: The Initial Darwinian Ancestor. Cold Spring Harb. Perspect. Biol..

[3] Cech T.R. (2012). The RNA Worlds in Context. Cold Spring Harb. Perspect. Biol..

[4] Kruger K., Grabowski P.J., Zaug A.J., Sands J., Gottschling D.E., Cech T.R. (1982). Self-splicing RNA: Autoexcision and Autocyclization of the Ribosomal RNA Intervening Sequence of Tetrahymena. Cell.

[5] Guerrier-Takada C., Gardiner K., Marsh T., Pace N., Altman S. (1983). The RNA Moiety of Ribonuclease P Is the Catalytic Subunit of the Enzyme. Cell.

[6] Miller S.L. (1953). A Production of Amino Acids Under Possible Primitive Earth Conditions. Science.

[7] Ferus M., Pietrucci F., Saitta A.M., Knizek A., Kubelik P., Ivanek O., Shestivska V., Civis S. (2017). Formation of Nucleobases in a Miller-Urey Reducing Atmosphere. Proc. Natl. Acad. Sci. USA.

[8] Fox G.E. (2010). Origin and Evolution of the Ribosome. Cold Spring Harb. Perspect. Biol..

[9] Bokov K., Steinberg S.V.A. (2009). Hierarchical Model for Evolution of 23S Ribosomal RNA. Nature.

[10] Bowman J.C., Petrov A.S., Frenkelpinter M., Penev P.I., Williams L.D. (2020). Root of the Tree: The Significance, Evolution, and Origins of the Ribosome. Chem. Rev..

[11] Yoon S.H., Ha S.M., Kwon S., Lim J., Kim Y., Seo H., Chun J. (2017). Introducing EzBioCloud: A Taxonomically United Database of 16S rRNA and Whole Genome Assemblies. Int. J. Syst. Evol. Microbiol..

[12] Petersen M., Meusemann K., Donath A., Dowling D., Liu S.L., Peters R.S., Podsiadlowski L., Vasilikopoulos A., Zhou X., Misof B. (2017). Orthograph: A Versatile Tool for Mapping Coding Nucleotide Sequences to Clusters of Orthologous Genes. BMC Bioinf..

[13] Zhu Q.Y., Mai U., Pfeiffer W., Janssen S., Asnicar F., Sanders J.G., Belda-Ferre P., Al-Ghalith G.A., Kopylova E., McDonald D. (2019). Phylogenomics of 10,575 Genomes Reveals Evolutionary Proximity Between Domains Bacteria and Archaea. Nat. Commun..

[14] Katoh K., Standley D.M. (2013). MAFFT Multiple Sequence Alignment Software Version 7: Improvements in Performance and Usability. Mol. Biol. Evol..

[15] Talavera G., Castresana J. (2007). Improvement of Phylogenies After Removing Divergent and Ambiguously Aligned Blocks from Protein Sequence Alignments. Syst. Biol..

[16] Vaidya G., Lohman D.J., Meier R. (2011). SequenceMatrix: Concatenation Software for the Fast Assembly of Multi-gene Datasets with Character Set and Codon Information. Cladistics.

[17] Nguyen L.T., Schmidt H.A., von Haeseler A., Minh B.Q. (2015). IQ-TREE: A Fast and Effective Stochastic Algorithm for Estimating Maximum-likelihood Phylogenies. Mol. Biol. Evol..

[18] Stamatakis A. (2014). RAxML Version 8: A Tool for Phylogenetic Analysis and Post-analysis of Large Phylogenies. Bioinformatics.

[19] Lemoine F., Entfellner J.B.D., Wilkinson E., Correia D., Felipe M.D., De Oliveira T., Gascuel O. (2018). Renewing Felsenstein’s Phylogenetic Bootstrap in the Era of Big Data. Nature.

[20] Rundlet E.J., Holm M., Schacherl M., Natchiar S.K., Altman R.B., Spahn C.M.T., Myasnikov A.G., Blanchard S.C. (2021). Structural Basis of Early Translocation Events on the Ribosome. Nature.

[21] Parks D.H., Chuvochina M., Chaumeil P.A., Rinke C., Mussig A.J., Hugenholtz P. (2020). A Complete Domain-to-species Taxonomy for Bacteria and Archaea. Nat. Biotechnol..

[22] Rinke C., Chuvochina M., Mussig A.J., Chaumeil P.A., Davin A.A., Waite D.W., Whitman W.B., Parks D.H., Hugenholtz P. (2021). A Standardized Archaeal Taxonomy for the Genome Taxonomy Database. Nat. Microbiol..

[23] Eme L., Spang A., Lombard J., Stairs C.W., Ettema T.J.G. (2017). Archaea and the Origin of Eukaryotes. Nat. Rev. Microbiol..

[24] Liu Y., Makarova K.S., Huang W.C., Wolf Y.I., Nikolskaya A.N., Zhang X.X., Cai M.W., Zhang C.J., Xu W., Luo Z.H. (2021). Expanded Diversity of Asgard Archaea and Their Relationships with Eukaryotes. Nature.

[25] Bashan A., Agmon I., Zarivach R., Schluenzen F., Harms J., Berisio R., Bartels H., Franceschi F., Auerbach T., Hansen H.A.S. (2003). Structural Basis of the Ribosomal Machinery for Peptide Bond Formation, Translocation, and Nascent Chain Progression. Mol. Cell.

[26] Doring T., Mitchell P., Osswald M., Bochkariov D., Brimacombe R. (1994). The Decoding Region of 16S RNA; A Cross-linking Study of the Ribosomal A, P and E Sites Using tRNA Derivatized at Position 32 in the Anticodon Loop. EMBO J..

[27] Green R., Noller H.F. (1997). Ribosomes and Translation. Annu. Rev. Biochem..

[28] Moazed D., Noller H.F. (1990). Binding of tRNA to the Ribosomal A and P Sites Protects Two Distinct Sets of Nucleotides in 16S rRNA. J. Mol. Biol..

[29] Nissen P., Ippolito J.A., Ban N., Moore P.B., Steitz T.A. (2001). RNA Tertiary Interactions in the Large Ribosomal Subunit: The A-minor Motif. Proc. Natl. Acad. Sci. USA.

[30] Polacek N., Mankin A.S. (2005). The Ribosomal Peptidyl Transferase Center: Structure, Function, Evolution, Inhibition. Crit. Rev. Biochem. Mol. Biol..

[31] Santer U.V., Cekleniak J., Kansil S., Santer M., O’Connor M., Dahlberg A.E. (1995). A Mutation at the Universally Conserved Position 529 in *Escherichia coli* 16S rRNA Creates a Functional but Highly Error Prone Ribosome. RNA.

[32] Yusupov M.M., Yusupova G.Z., Baucom A., Lieberman K., Earnest T.N., Cate J.H.D., Noller H.F. (2001). Crystal Structure of the Ribosome at 5.5 Resolution. Science.

[33] Zarivach R., Bashan A., Berisio R., Harms J., Auerbach T., Schluenzen F., Bartels H., Baram D., Pyetan E., Sittner A. (2004). Functional Aspects of Ribosomal Architecture: Symmetry, Chirality and Regulation. Phys. Org. Chem..

[34] Bhangu R., Wollenzien P. (1992). The mRNA Binding Track in the *Escherichia coli* Ribosome for mRNAs of Different Sequences. Biochemistry.

[35] Bhangu R., Juzumiene D., Wollenzien P. (1994). Arrangement of Messenger RNA on *Escherichia coli* Ribosomes with Respect to 10 16S rRNA Cross-linking Sites. Biochemistry.

[36] Juzumiene D.I., Shapkina T.G., Wollenzien P. (1995). Distribution of Cross-links between mRNA Analogues and 16S rRNA in *Escherichia coli* 70S Ribosomes Made Under Equilibrium Conditions and Their Response to tRNA Binding. J. Biol. Chem..

[37] Rinke-Appel J., Junke N., Brimacombe R., Dukudovskaya S., Dontsova O., Bogdanov A. (1993). Site-directed Cross-linking of mRNA Analogues to 16S Ribosomal RNA; A Complete Scan of Cross-links from All Positions Between ‘+1’ and ‘+16’ on the mRNA, Downstream from the Decoding Site. Nucleic Acids Res..

[38] Ogle J.M., Brodersen D.E., Clemons W.M., Tarry M.J., Carter A.P., Ramakrishnan V. (2001). Recognition of Cognate Transfer RNA by the 30S Ribosomal Subunit. Science.

[39] Huttenhofer A., Noller H.F. (1994). Footprinting mRNA-ribosome Complexes with Chemical Probes. EMBO J..

[40] Stade K., Riens S., Bochkariov D., Brimacombe R. (1994). Contacts between the Growing Peptide Chain and the 23S RNA in the 50S Ribosomal Subunit. Nucleic Acids Res..

[41] Agmon I., Amit M., Auerbach T., Bashan A., Baram D., Bartels H., Berisio R., Greenberg I., Harms J., Hansen H.A.S. (2004). Ribosomal Crystallography: A Flexible Nucleotide Anchoring tRNA Translocation, Facilitates Peptide-bond Formation, Chirality Discrimination and Antibiotics Synergism. FEBS Lett..

[42] Bashan A., Zarivach R., Schluenzen F., Agmon I., Harms J., Auerbach T., Baram D., Berisio R., Bartels H., Hansen H.A.S. (2003). Ribosomal Crystallography: Peptide Bond Formation and Its Inhibition. Biopolymers.

[43] Koch M., Willi J., Pradere U., Hall J., Polacek N. (2017). Critical 23S rRNA Interactions for Macrolide-dependent Ribosome Stalling on the ErmCL Nascent Peptide Chain. Nucleic Acids Res..

[44] O’Connor M., Goringer H.U., Dahlberg A.E. (1992). A Ribosomal Ambiguity Mutation in the 530 Loop of *E. coli* 16S rRNA. Nucleic Acids Res..

[45] Yonath A. (2005). Antibiotics Targeting Ribosomes: Resistance, Selectivity, Synergism, and Cellular Regulation. Annu. Rev. Biochem..

[46] Yoshizawa S., Fourmy D., Puglisi J.D. (1999). Recognition of the Codon-anticodon Helix by Ribosomal RNA. Science.

[47] Schmeing T.M., Huang K.S., Strobel S.A., Steitz T.A. (2005). An Induced-fit Mechanism to Promote Peptide Bond Formation and Exclude Hydrolysis of Peptidyl-tRNA. Nature.

[48] Dedkova L.M., Hecht S.M. (2019). Expanding the Scope of Protein Synthesis Using Modified Ribosomes. J. Am. Chem. Soc..

[49] Voorhees R.M., Weixlbaumer A., Loakes D., Kelley A.C., Ramakrishnan V. (2009). Insights into Substrate Stabilization from Snapshots of the Peptidyl Transferase Center of the Intact 70S Ribosome. Nat. Struct. Mol. Biol..

[50] Gao H.X., Sengupta J., Valle M., Korostelev A., Eswar N., Stagg S.M., Van Roey P., Agrawal R.K., Harvey S.C., Sali A. (2003). Study of the Structural Dynamics of the *E. coli* 70S Ribosome Using Real-space Refinement. Cell.

[51] Schmeing T.M., Ramakrishnan V. (2009). What Recent Ribosome Structures Have Revealed About the Mechanism of Translation. Nature.

[52] Poehlsgaard J., Douthwaite S. (2005). The Bacterial Ribosome as a Target for Antibiotics. Nat. Rev. Microbiol..

[53] Allen G.S., Zavialov A., Gursky R., Ehrenberg M., Frank J. (2005). The Cryo-EM Structure of a Translation Initiation Complex from *Escherichia coli*. Cell.

[54] Klaholz B.P., Myasnikov A.G., Van Heel M. (2004). Visualization of Release Factor 3 on the Ribosome During Termination of Protein Synthesis. Nature.

[55] Moazed D., Robertson J.M., Noller H.F. (1988). Interaction of elongation factors EF-G and EF-Tu with a conserved loop in 23S RNA. Nature.

[56] Stark H., Stark H., Rodnina M.V., Wieden H.J., Zemlin F., Wintermeyer W., van Heel M. (2002). Ribosome Interactions of Aminoacyl-tRNA and Elongation Factor Tu in the Codon-recognition Complex. Nat. Struct. Biol..

[57] Valle M., Zavialov A., Li W., Stagg S.M., Sengupta J., Nielsen R.C., Nissen P., Harvey S.C., Ehrenberg M., Frank J. (2003). Incorporation of Aminoacyl-tRNA into the Ribosome as Seen by Cryo-electron Microscopy. Nat. Struct. Biol..

[58] Valle M., Zavialov A., Sengupta J., Rawat U., Ehrenberg M., Frank J. (2003). Locking and Unlocking of Ribosomal Motions. Cell.

[59] Crick F.H.C. (1968). The Origin of the Genetic Code. J. Mol. Biol..

[60] Hug L.A., Baker B.J., Anantharaman K., Brown C.T., Probst A.J., Castelle C.J., Butterfield C.N., Hernsdorf A.W., Amano Y., Ise K. (2016). A New View of the Tree of Life. Nat. Microbiol..

[61] Williams T.A., Cox C.J., Foster P.G., Szollosi G.J., Embley T.M. (2020). Phylogenomics Provides Robust Support for A Two-domains Tree of Life. Nat. Ecol. Evol..

[62] Martínez Giménez J.A., Sáez G.T., Seisdedos R.T. (1998). On the Function of Modified Nucleosides in the RNA World. J. Theor. Biol..

[63] Davidovich C., Belousoff M., Wekselman I., Shapira T., Krupkin M., Zimmerman E., Bashan A., Yonath A. (2010). The Proto-ribosome: An Ancient Nano-machine for Peptide Bond Formation. Isr. J. Chem..

[64] Huang L.L., Krupkin M., Bashan A., Yonath A., Massa L. (2013). Protoribosome by Quantum Kernel Energy Method. Proc. Natl. Acad. Sci. USA.

[65] Agmon I.C. (2016). Could a Proto-ribosome Emerge Spontaneously in the Prebiotic World?. Molecules.

[66] Petrov A.S., Bernier C.R., Hsiao C.L., Norris A.M., Kovacs N.A., Waterbury C.C., Stepanov V.G., Harvey S.C., Fox G.E., Wartell R.M. (2014). Evolution of the Ribosome at Atomic Resolution. Proc. Natl. Acad. Sci. USA.

[67] Petrov A.S., Gulen B., Norris A.M., Kovacs N.A., Bernier C.R., Lanier K.A. (2015). History of the Ribosome and the Origin of Translation. Proc. Natl. Acad. Sci. USA.

