# Supplementary material for: Reconstruction of the rRNA Sequences of LUCA, with Bioinformatic Implication of the Local Similarities Shared by Them
Source: Biology (Basel). 2022 May 29;11(6):837. doi: 10.3390/biology11060837 (PMC9219793; doi:10.3390/biology11060837)
Supplement: Supplementary file 1 [file biology-11-00837-s001.zip › Table S1.pdf]

Supplementary Table S1. Information of the sampled species.

Domain	Code name of species	Phylum	Species	Accession number
Archaea	Aenigmarchaeota-3-1	Aenigmarchaeota	AB722182_s	/
Archaea	Aenigmarchaeota-3-2	Aenigmarchaeota	AB722186_s	/
Archaea	Aenigmarchaeota-3-3	Aenigmarchaeota	Candidatus Aenigmarchaeota archaeon CG1_02_38_14	ASM187398v1
Archaea	Crenarchaeota-5-1	Crenarchaeota	*Caldisphaera lagunensis*	ASM31779v1
Archaea	Crenarchaeota-5-2	Crenarchaeota	*Pyrolobus fumarii*	ASM22339v1
Archaea	Crenarchaeota-5-3	Crenarchaeota	*Fervidicoccus fontis*	ASM25842v1
Archaea	Crenarchaeota-5-4	Crenarchaeota	*Pyrobaculum oguniense*	ASM24754v1
Archaea	Crenarchaeota-5-5	Crenarchaeota	*Ignisphaera aggregans*	ASM14598v1
Archaea	Euryarchaeota-13-1	Euryarchaeota	*Archaeoglobus profundus*	ASM2528v1
Archaea	Euryarchaeota-13-2	Euryarchaeota	*Halobacterium salinarum*	ASM6902v1
Archaea	Euryarchaeota-13-3	Euryarchaeota	*Halobiforma lacisalsi*	ASM33665v1
Archaea	Euryarchaeota-13-4	Euryarchaeota	*Methanobacterium lacus*	ASM19158v1
Archaea	Euryarchaeota-13-5	Euryarchaeota	*Methanocaldococcus fervens*	ASM2398v1
Archaea	Euryarchaeota-13-6	Euryarchaeota	*Methanocella paludicola*	ASM1100v1
Archaea	Euryarchaeota-13-7	Euryarchaeota	*Methanoculleus bourgensis*	Mb_MS2
Archaea	Euryarchaeota-13-8	Euryarchaeota	*Methanohalophilus mahii*	ASM2586v1
Archaea	Euryarchaeota-13-9	Euryarchaeota	*Methanopyrus kandleri*	ASM718v1
Archaea	Euryarchaeota-13-10	Euryarchaeota	*Pyrococcus abyssi*	ASM19593v1
Archaea	Euryarchaeota-13-11	Euryarchaeota	*Methanomethylophilus alvus*	ASM30025v2
Archaea	Euryarchaeota-13-12	Euryarchaeota	*Picrophilus torridus*	ASM826v1
Archaea	Euryarchaeota-13-13	Euryarchaeota	*Methanothermus fervidus*	ASM16609v1
Archaea	Euryarchaeota-14	Euryarchaeota	*Archaeoglobus sulfaticallidus*	ASM38556v1
Archaea	Euryarchaeota-15	Euryarchaeota	*Geoglobus ahangari*	ASM100604v1

Domain	Code name of species	Phylum	Species	Accession number
Archaea	Euryarchaeota-16	Euryarchaeota	*Halobiforma haloterrestris*	IMG-taxon 2693429869 annotated assembly
Archaea	Euryarchaeota-17	Euryarchaeota	*Methanotorris formicicus*	ASM24345v2
Archaea	Euryarchaeota-18	Euryarchaeota	*Methanocaldococcus bathoardescens*	ASM73906v1
Archaea	Euryarchaeota-19	Euryarchaeota	*Palaeococcus pacificus*	ASM72542v1
Archaea	Euryarchaeota-20	Euryarchaeota	*Thermococcus chitonophagus*	Pyrococcus chitonophagus genome sequence
Archaea	Euryarchaeota-21	Euryarchaeota	*Thermogymnomonas acidicola*	ASM131610v1
Archaea	Euryarchaeota-22	Euryarchaeota	*Methanobrevibacter ruminantium*	ASM2418v1
Archaea	Euryarchaeota-25	Euryarchaeota	*Methanonatronarchaeum thermophilum*	ASM215391v1
Archaea	Euryarchaeota-26	Euryarchaeota	Methanonatronarchaeia archaeon	ASM421203v1
Archaea	Euryarchaeota-27	Euryarchaeota	Candidatus *Hadarchaeum yellowstonense*	ASM151520v2
Archaea	Euryarchaeota-28	Euryarchaeota	Hadesarchaea archaeon	ASM1436109v1
Archaea	Euryarchaeota-29	Euryarchaeota	Candidatus *Thalassarchaeum betae*	ASM319392v1
Archaea	Euryarchaeota-31	Euryarchaeota	Candidatus *Methanoliparum thermophilum*	ASM421207v1
Archaea	Euryarchaeota-32	Euryarchaeota	Candidatus *Methanolliviera hydrocarbonicum*	ASM421208v1
Archaea	Euryarchaeota-33	Euryarchaeota	candidate divison MSBL1 archaeon SCGC-AAA259A05	SCGC-AAA259A05
Archaea	Euryarchaeota-36	Euryarchaeota	candidate divison MSBL1 archaeon SCGC-AAA259O05	SCGC-AAA259O05
Archaea	Euryarchaeota-37	Euryarchaeota	candidate divison MSBL1 archaeon SCGC-AAA261D19	SCGC-AAA261D19
Archaea	Euryarchaeota-38	Euryarchaeota	candidate divison MSBL1 archaeon SCGC-AAA382A20	SCGC-AAA382A20
Archaea	Euryarchaeota-39	Euryarchaeota	candidate divison MSBL1 archaeon SCGC-AAA382K21	SCGC-AAA382K21
Archaea	Euryarchaeota-41	Euryarchaeota	candidate divison MSBL1 archaeon SCGC-AAA382C18	SCGC-AAA382C18
Archaea	Euryarchaeota-42	Euryarchaeota	candidate divison MSBL1 archaeon SCGC-AAA385D11	SCGC-AAA385D11
Archaea	Euryarchaeota-44	Euryarchaeota	*Aciduliprofundum boonei*	ASM15108v1
Archaea	Euryarchaeota-45	Euryarchaeota	uncultured marine group II euryarchaeote	ASM24673v1
Archaea	Euryarchaeota-46	Euryarchaeota	*Aciduliprofundum* sp.	ASM32750v1
Archaea	Euryarchaeota-53	Euryarchaeota	Euryarchaeota archaeon SG8-5	ASM159588v1

Domain	Code name of species	Phylum	Species	Accession number
Archaea	Euryarchaeota-55	Euryarchaeota	Euryarchaeota archaeon CG_4_9_14_3_um_filter_38_12	ASM279005v1
Archaea	Euryarchaeota-56	Euryarchaeota	Euryarchaeota archaeon TMED164	ASM217093v1
Archaea	Euryarchaeota-57	Euryarchaeota	Euryarchaeota archaeon ADurb.Bin023	ASM207130v1
Archaea	Euryarchaeota-58	Euryarchaeota	candidate division MSBL1 archaeon SCGC-AAA259D14	SCGC-AAA259D14
Archaea	Euryarchaeota-59	Euryarchaeota	candidate division MSBL1 archaeon SCGC-AAA259E17	SCGC-AAA259E17
Archaea	Euryarchaeota-60	Euryarchaeota	Candidatus Altiarchaeales archaeon WOR_SM1_79	ASM172383v1
Archaea	Euryarchaeota-61	Euryarchaeota	Candidatus Altiarchaeales archaeon WOR_SM1_SCG	ASM172384v1
Archaea	Euryarchaeota-62	Euryarchaeota	Candidatus Altiarchaeales archaeon WOR_SM1_86-2	ASM172385v1
Archaea	Euryarchaeota-63	Euryarchaeota	Candidatus Altiarchaeales archaeon IMC4 isolate IMC4_SM1	ASM174278v1
Archaea	Euryarchaeota-73	Euryarchaeota	Candidatus *Altiarchaeum* sp. CG2_30_32_3053	ASM187384v1
Archaea	Euryarchaeota-78	Euryarchaeota	Euryarchaeota archaeon HMET1	ASM191440v1
Archaea	Euryarchaeota-90	Euryarchaeota	Euryarchaeota archaeon TMED85	ASM217036v2
Archaea	Euryarchaeota-97	Euryarchaeota	*Aciduliprofundum boonei*	ASM2566v1
Archaea	Korarchaeota-3-1	Korarchaeota	*Korarchaeum cryptofilum*	ASM1960v1
Archaea	Korarchaeota-3-2	Korarchaeota	AM749964_s	/
Archaea	Korarchaeota-3-3	Korarchaeota	DQ228520_s	/
Archaea	Nanoarchaeota-2-1	Nanoarchaeota	*Nanoarchaeum equitans*	ASM808v1
Archaea	Nanoarchaeota-2-2	Nanoarchaeota	Candidatus *Nanopusillus acidilobi*	ASM155201v1
Archaea	Thaumarchaeota-4-1	Thaumarchaeota	*Nitrosopelagicus brevis*	ASM81218v1
Archaea	Thaumarchaeota-4-2	Thaumarchaeota	*Nitrosoarchaeum limnia*	ASM20458v1
Archaea	Thaumarchaeota-4-3	Thaumarchaeota	*Nitrososphaera viennensis*	ASM69878v1
Archaea	Thaumarchaeota-4-4	Thaumarchaeota	*Cenarchaeum symbiosum*	ASM20071v1
Archaea	Candidatus-Bathyarchaeota-3-1	Candidatus Bathyarchaeota	Candidatus Bathyarchaeota archaeon BA2	ASM139979v1
Archaea	Candidatus-Bathyarchaeota-3-2	Candidatus Bathyarchaeota	candidatus_bathyarchaeota_archaeon_ba1	ASM139980v1
Archaea	Candidatus-Bathyarchaeota-3-3	Candidatus Bathyarchaeota	Candidatus Bathyarchaeota archaeon RBG_16_48_13	ASM177599v1

Domain	Code name of species	Phylum	Species	Accession number
Archaea	Candidatus-Odinarchaeota-1-1	Candidatus Odinarchaeota	Candidatus Odinarchaeota archaeon LCB_4	ASM194066v1
Archaea	Candidatus-Thorarchaeota-3-1	Candidatus Thorarchaeota	Candidatus Thorarchaeota archaeon SMTZ1-83	ASM156332v1
Archaea	Candidatus-Thorarchaeota-3-2	Candidatus Thorarchaeota	Candidatus Thorarchaeota archaeon SMTZ1-45	ASM156333v1
Archaea	Candidatus-Thorarchaeota-3-3	Candidatus Thorarchaeota	Candidatus Thorarchaeota archaeon	ASM366276v1
Archaea	Candidatus-Verstraetearchaeota-2-1	Candidatus Verstraetearchaeota	Candidatus *Methanomethylicus mesodigestum*	ASM171700v1
Archaea	Candidatus-Verstraetearchaeota-2-2	Candidatus Verstraetearchaeota	Candidatus *Methanomethylicus oleusabulum*	ASM171702v1
Archaea	Candidatus-Woesearchaeota-1-1	Candidatus Woesearchaeota	Candidatus Woesearchaeota archaeon CG1_02_57_44	ASM187141v1
Archaea	Unknown-8-1	environmental samples	halophilic archaeon DL31	ASM22447v1
Archaea	Unknown-8-2	environmental samples	archaeon GW2011_AR16	ASM80592v1
Archaea	Unknown-8-3	environmental samples	uncultured archaeon A07HB70	A07HB70_072612b_select
Archaea	Unknown-8-4	environmental samples	uncultured archaeon A07HN63	A07HN63_021313_select
Archaea	Unknown-8-5	environmental samples	uncultured archaeon A07HR60	A07HR60_111512_select
Archaea	Unknown-8-6	environmental samples	archaeon GW2011_AR10	ASM83027v1
Archaea	Unknown-8-7	environmental samples	archaeon GW2011_AR15	ASM83029v1
Archaea	Unknown-8-8	environmental samples	uncultured archaeon	ASM96574v1
Archaea	Candidatus-Heimdallarchaeota-2-1	Candidatus Heimdallarchaeota	Candidatus Heimdallarchaeota archaeon LC_3	ASM194064v1
Archaea	Candidatus-Heimdallarchaeota-2-2	Candidatus Heimdallarchaeota	Candidatus Heimdallarchaeota archaeon AB_125	ASM194075v1
Archaea	Candidatus-Heimdallarchaeota-3-3	Candidatus Heimdallarchaeota	Candidatus Heimdallarchaeota archaeon B3_Heim	ASM314427v1
Archaea	Candidatus-Heimdallarchaeota-4	Candidatus Heimdallarchaeota	Candidatus Heimdallarchaeota archaeon LC_2	ASM194072v1
Archaea	Candidatus-Diapherotrites-3-1	Candidatus Diapherotrites	Candidatus *Iainarchaeum andersonii*	ASM40235v1
Archaea	Candidatus-Diapherotrites-3-2	Candidatus Diapherotrites	Diapherotrites archaeon SCGC AAA011-N19	ASM40452v1
Archaea	Candidatus-Diapherotrites-3-3	Candidatus Diapherotrites	Diapherotrites archaeon SCGC AAA011-K09	ASM40454v1
Archaea	Candidatus-Lokiarchaeota-3-1	Candidatus Lokiarchaeota	*Lokiarchaeum* sp.	ASM98684v1
Archaea	Candidatus-Lokiarchaeota-3-2	Candidatus Lokiarchaeota	Anaerobic archaeon MK-D1	ASM800077v1
Archaea	Candidatus-Lokiarchaeota-3-3	Candidatus Lokiarchaeota	Candidatus Lokiarchaeota archaeon	ASM366286

Domain	Code name of species	Phylum	Species	Accession number
Archaea	Candidatus-Micrarchaeota-3-1	Candidatus Micrarchaeota	Candidatus Micrarchaeota archaeon CG1_02_47_40	ASM187147v1
Archaea	Candidatus-Micrarchaeota-3-2	Candidatus Micrarchaeota	Candidatus *Micrarchaeum acidiphilum*	ASM188998v1
Archaea	Candidatus-Micrarchaeota-3-3	Candidatus Micrarchaeota	Candidatus *Micrarchaeum* sp.	ASM189651v1
Archaea	Candidatus-Nanohaloarchaeota-3-1	Candidatus Nanohaloarchaeota	Candidatus *Haloredivivus* sp.	ASM23619v1
Archaea	Candidatus-Nanohaloarchaeota-3-2	Candidatus Nanohaloarchaeota	Nanohaloarchaea archaeon SG9	ASM176142v1
Archaea	Candidatus-Nanohaloarchaeota-3-3	Candidatus Nanohaloarchaeota	Candidatus *Nanosalinarum* sp.	ASM22035v1
Archaea	Candidatus-Geothermarchaeota-1-1	Candidatus Geothermarchaeota	Candidatus Geothermarchaeota archaeon JdFR-14	ASM201108v1
Archaea	Candidatus-Pacearchaeota-3-1	Candidatus Pacearchaeota	Candidatus Pacearchaeota archaeon RBG_19FT_COMBO_34_9	ASM178641v1
Archaea	Candidatus-Pacearchaeota-3-2	Candidatus Pacearchaeota	Candidatus Pacearchaeota archaeon RBG_16_35_8	ASM178642v1
Archaea	Candidatus-Pacearchaeota-3-3	Candidatus Pacearchaeota	Candidatus Pacearchaeota archaeon CG1_02_31_27	ASM187214v1
Bacteria	Candidatus-Bipolaricaulota-3-1	Candidatus Bipolaricaulota	Acetothermia bacterium SCGC AAA255-C06	ASM40496v1
Bacteria	Candidatus-Bipolaricaulota-3-2	Candidatus Bipolaricaulota	Acetothermia bacterium 64_32 ASM150815v1	ASM150815v1
Bacteria	Candidatus-Bipolaricaulota-3-3	Candidatus Bipolaricaulota	Candidatus Acetothermia bacterium JdFR-46	ASM201136v1
Bacteria	Acidobacteria-3-1	Acidobacteria	*Acidobacterium capsulatum*	ASM2256v1
Bacteria	Acidobacteria-3-2	Acidobacteria	*Pyrinomonas methylaliphatogenes*	K22
Bacteria	Acidobacteria-3-3	Acidobacteria	*Solibacter usitatus*	ASM1490v1
Bacteria	Actinobacteria-4-1	Actinobacteria	*Eggerthella lenta*	ASM2426v1
Bacteria	Actinobacteria-4-2	Actinobacteria	*Rubrobacter xylanophilus*	ASM1418v1
Bacteria	Actinobacteria-4-3	Actinobacteria	*Conexibacter woesei*	ASM2526v1
Bacteria	Actinobacteria-4-4	Actinobacteria	*Rubrobacter radiotolerans*	ASM66189v1
Bacteria	Candidatus-Aerophobetes-2-1	Candidatus Aerophobetes	Aerophobetes bacterium JGI 0000014-A15	ASM40436v1
Bacteria	Candidatus-Aerophobetes-2-2	Candidatus Aerophobetes	Aerophobetes bacterium SCGC AAA255-F10	ASM40434v1
Bacteria	Candidatus-Aminicenantes-2-1	Candidatus Aminicenantes	Aminicenantes bacterium SCGC AAA252-D18	ASM40440v1
Bacteria	Candidatus-Aminicenantes-2-2	Candidatus Aminicenantes	Aminicenantes bacterium SCGC AAA252-P19	ASM40528v1
Bacteria	Aquificae-3-1	Aquificae	*Thermocrinis ruber*	ASM51273v1

Domain	Code name of species	Phylum	Species	Accession number
Bacteria	Aquificae-3-2	Aquificae	*Sulfurihydrogenibium azorense*	ASM2154v1
Bacteria	Aquificae-3-3	Aquificae	*Thermovibrio ammonificans*	ASM18580v1
Bacteria	Armatimonadetes-3-1	Armatimonadetes	*Chthonomonas calidirosea*	T49
Bacteria	Armatimonadetes-3-2	Armatimonadetes	*Fimbriimonas ginsengisoli*	ASM72462v1
Bacteria	Armatimonadetes-3-3	Armatimonadetes	Armatimonadetes bacterium 55-13	ASM189803v1
Bacteria	Atribacterota-3-1	Atribacterota	Candidatus Atribacteria bacterium RBG_19FT_COMBO_35_14	ASM177395v1
Bacteria	Atribacterota-5	Atribacterota	Candidatus Atribacteria bacterium 1244-E10-H5-B2	ASM357524v1
Bacteria	Atribacterota-8	Atribacterota	Candidatus Atribacteria bacterium CG_4_10_14_3_um_filter_34_13	ASM278428v1
Bacteria	Atribacterota-9	Atribacterota	Candidatus Atribacteria bacterium CG_4_8_14_3_um_filter_34_18	ASM278267v1
Bacteria	Atribacterota-13	Atribacterota	Candidatus Atribacteria bacterium ADurb.Bin276	ASM206960v1
Bacteria	Atribacterota-15	Atribacterota	Atribacteria bacterium 34_128	ASM150928v1
Bacteria	Atribacterota-16	Atribacterota	Atribacteria bacterium 34_868	ASM150865v1
Bacteria	Bacteroidetes-3-1	Bacteroidetes	*Chitinophaga pinensis*	ASM2400v1
Bacteria	Bacteroidetes-3-2	Bacteroidetes	*Coprobacter secundus*	ASM80310v1
Bacteria	Bacteroidetes-3-3	Bacteroidetes	*Fluviicola taffensis*	ASM19460v1
Bacteria	Candidatus-Berkelbacteria-3-1	Candidatus Berkelbacteria	Berkelbacteria bacterium GW2011_GWA2_35_9	ASM99118v1
Bacteria	Candidatus-Berkelbacteria-3-2	Candidatus Berkelbacteria	Berkelbacteria bacterium GW2011_GWA2_38_9	ASM99313v1
Bacteria	Candidatus-Berkelbacteria-3-3	Candidatus Berkelbacteria	Candidatus Berkelbacteria bacterium RIFCSPLOWO2_01_FULL_50_28	ASM177439v1
Bacteria	Caldiserica-3-1	Caldiserica	*Caldisericum exile*	ASM28433v1
Bacteria	Caldiserica-3-2	Caldiserica	AB252429_s	/
Bacteria	Caldiserica-3-3	Caldiserica	AB364883_s	/
Bacteria	Calditrichaeota-3	Calditrichaeota	*Caldithrix abyssi*	/
Bacteria	Calditrichaeota-4	Caldithrix p	AF317742_s	/
Bacteria	Candidatus-Calescamantes-2-1	Candidatus Calescamantes	Calescamantes bacterium JGI 0000106-J16	ASM40466v1
Bacteria	Candidatus-Calescamantes-2-2	Candidatus Calescamantes	Calescamantes bacterium JGI 0000106-N7	ASM40468v1

Domain	Code name of species	Phylum	Species	Accession number
Bacteria	Chlamydiae-3-1	Chlamydiae	*Simkania negevensis*	ASM23720v1
Bacteria	Chlamydiae-3-2	Chlamydiae	*Criblamydia sequanensis*	CS_CRIB18-1
Bacteria	Chlamydiae-3-3	Chlamydiae	*Waddlia chondrophila*	ASM161826v1
Bacteria	Chlorobi-2-1	Chlorobi	*Chloroherpeton thalassium*	ASM2052v1
Bacteria	Chlorobi-2-2	Chlorobi	*Prosthecochloris aestuarii*	ASM2062v1
Bacteria	Chloroflexi-6-1	Chloroflexi	*Caldilinea aerophila*	ASM28117v1
Bacteria	Chloroflexi-6-2	Chloroflexi	*Chloroflexus aggregans*	ASM2194v1
Bacteria	Chloroflexi-6-3	Chloroflexi	*Herpetosiphon aurantiacus*	ASM1856v1
Bacteria	Chloroflexi-6-4	Chloroflexi	*Dehalococcoides mccartyi*	ASM2502v1
Bacteria	Chloroflexi-6-5	Chloroflexi	*Thermogemmatispora onikobensis*	ASM174828v1
Bacteria	Chloroflexi-6-6	Chloroflexi	*Nitrolancea hollandica*	ASM29725v1
Bacteria	Chrysiogenetes-3-1	Chrysiogenetes	*Chrysiogenes arsenatis*	Chrysiogenes arsenatis
Bacteria	Chrysiogenetes-3-2	Chrysiogenetes	*Desulfurispirillum indicum*	ASM17763v2
Bacteria	Chrysiogenetes-3-3	Chrysiogenetes	*Desulfurispirillum alkaliphilum*	/
Bacteria	Cloacimonetes-2-1	Cloacamonas p	*Cloacamonas acidaminovorans*	/
Bacteria	Cloacimonetes-2-2	Cloacamonas p	AJ347760_s	/
Bacteria	Cyanobacteria-7-1	Cyanobacteria	*Rubidibacter lacunae*	KS51_v1
Bacteria	Cyanobacteria-7-2	Cyanobacteria	*Chamaesiphon minutus*	ASM31714v1
Bacteria	Cyanobacteria-7-3	Cyanobacteria	*Crinalium epipsammum*	ASM31749v1
Bacteria	Cyanobacteria-7-4	Cyanobacteria	*Gloeobacter violaceus*	ASM1138v1
Bacteria	Cyanobacteria-7-5	Cyanobacteria	*Gloeomargarita lithophora*	ASM187022v1
Bacteria	Cyanobacteria-7-6	Cyanobacteria	*Nostoc punctiforme*	ASM2002v1
Bacteria	Cyanobacteria-7-7	Cyanobacteria	*Fischerella major*	ASM190464v1
Bacteria	Deferribacteres-3-1	Deferribacteres	*Denitrovibrio acetiphilus*	ASM2572v1
Bacteria	Deferribacteres-3-2	Deferribacteres	*Calditerrivibrio nitroreducens*	ASM18340v1

Domain	Code name of species	Phylum	Species	Accession number
Bacteria	Deferribacteres-3-3	Deferribacteres	*Flexistipes sinusarabici*	ASM21862v1
Bacteria	Deinococcus-Thermus-3-1	Deinococcus-Thermus	*Truepera radiovictrix*	ASM9242v1
Bacteria	Deinococcus-Thermus-3-2	Deinococcus-Thermus	*Deinococcus deserti*	ASM2068v1
Bacteria	Deinococcus-Thermus-3-3	Deinococcus-Thermus	*Thermus thermophilus*	ASM9154v1
Bacteria	Dictyoglomi-2-1	Dictyoglomi	*Dictyoglomus thermophilum*	ASM2096v1
Bacteria	Dictyoglomi-2-2	Dictyoglomi	*Dictyoglomus turgidum*	ASM2164v1
Bacteria	Elusimicrobia-3-1	Elusimicrobia	Elusimicrobia bacterium RIFOXYB12_FULL_50_12	ASM180045v1
Bacteria	Elusimicrobia-3-2	Elusimicrobia	*Endomicrobium proavitum*	ASM102754v1
Bacteria	Elusimicrobia-3-3	Elusimicrobia	*Elusimicrobium minutum*	ASM2014v1
Bacteria	Candidatus-Fervidibacteria-2-1	Candidatus Fervidibacteria	*Fervidibacter sacchari*	Fervidibacteria bacterium OTU 1
Bacteria	Candidatus-Fervidibacteria-2-2	Candidatus Fervidibacteria	Fervidibacteria bacterium SCGC AAA471-D06	ASM38072v1
Bacteria	Fibrobacteres-3-1	Fibrobacteres	*Chitinispirillum alkaliphilum*	ASM104552v1
Bacteria	Fibrobacteres-3-2	Fibrobacteres	*Chitinivibrio alkaliphilus*	Acht1_1
Bacteria	Fibrobacteres-3-3	Fibrobacteres	*Fibrobacter succinogenes*	ASM2466v1
Bacteria	Firmicutes-12-1	Firmicutes	*Bacillus cereus*	ASM782v1
Bacteria	Firmicutes-12-2	Firmicutes	*Streptococcus suis*	ASM2674v1
Bacteria	Firmicutes-12-3	Firmicutes	*Eubacterium limosum*	ASM15224v2
Bacteria	Firmicutes-12-4	Firmicutes	*Halothermothrix orenii*	ASM2048v1
Bacteria	Firmicutes-12-5	Firmicutes	*Natranaerobius thermophilus*	ASM2000v1
Bacteria	Firmicutes-12-6	Firmicutes	*Desulforudis audaxviator*	ASM1842v1
Bacteria	Firmicutes-12-7	Firmicutes	*Limnochorda pilosa*	ASM154401v1
Bacteria	Firmicutes-12-8	Firmicutes	*Acidaminococcus fermentans*	IMG-taxon 2654588173 annotated assembly
Bacteria	Firmicutes-12-9	Firmicutes	*Pelosinus fermentans*	ASM27166v2
Bacteria	Firmicutes-12-10	Firmicutes	*Veillonella parvula*	ASM155333v1
Bacteria	Firmicutes-12-11	Firmicutes	*Holdemanella biformis*	ASM15665v1

Domain	Code name of species	Phylum	Species	Accession number
Bacteria	Firmicutes-12-12	Firmicutes	*Finegoldia magna*	ASM1018v1
Bacteria	Fusobacteria-3-1	Fusobacteria	*Fusobacterium nucleatum*	ASM732v1
Bacteria	Fusobacteria-3-2	Fusobacteria	*Streptobacillus moniliformis*	ASM2456v1
Bacteria	Fusobacteria-3-3	Fusobacteria	*Ilyobacter polytropus*	ASM16550v1
Bacteria	Gemmatimonadetes-3-1	Gemmatimonadetes	*Gemmatimonas aurantiaca*	ASM1030v1
Bacteria	Gemmatimonadetes-3-2	Gemmatimonadetes	*Gemmatirosa kalamazoonesis*	ASM52298v1
Bacteria	Gemmatimonadetes-3-3	Gemmatimonadetes	*Gemmatimonas phototrophica*	ASM69509v2
Bacteria	Candidatus-Hydrogenedentes-2-1	Candidatus Hydrogenedentes	*Hydrogenedens terephthalicus*	Hydrogenedentes bacterium OTU 1
Bacteria	Candidatus-Hydrogenedentes-2-2	Candidatus Hydrogenedentes	Hydrogenedentes bacterium JGI 0000039-J10	ASM38078v1
Bacteria	Kazan-2-1	Kazan	AM997374_s	/
Bacteria	Kazan-2-2	Kazan	AB637068_s	/
Bacteria	Candidatus-Latescibacteria-3-1	Candidatus Latescibacteria	Latescibacteria bacterium SCGC AAA257-K07	ASM40243v1
Bacteria	Candidatus-Latescibacteria-3-2	Candidatus Latescibacteria	Candidatus Latescibacter anaerobius	ASM40303v1
Bacteria	Candidatus-Latescibacteria-3-3	Candidatus Latescibacteria	Latescibacteria bacterium DG_63	ASM130290v1
Bacteria	Lentisphaerae-3-1	Lentisphaerae	*Lentisphaera araneosa*	ASM17075v1
Bacteria	Lentisphaerae-3-2	Lentisphaerae	Lentisphaerae bacterium GWF2_44_16	ASM180322v1
Bacteria	Lentisphaerae-3-3	Lentisphaerae	Lentisphaerae bacterium RIFOXYA12_64_32	ASM180323v1
Bacteria	Candidatus-Marinimicrobia-3-1	Candidatus Marinimicrobia	Marinimicrobia bacterium 46_47	ASM150835v1
Bacteria	Candidatus-Marinimicrobia-3-2	Candidatus Marinimicrobia	Marinimicrobia bacterium 46_43	ASM150933v1
Bacteria	Candidatus-Marinimicrobia-3-3	Candidatus Marinimicrobia	Candidatus Marinimicrobia bacterium CG1_02_48_14	ASM187268v1
Bacteria	Candidatus-Microgenomates-2-1	Candidatus Microgenomates	Microgenomates bacterium OLB23	ASM156724v1
Bacteria	Candidatus-Microgenomates-2-2	Candidatus Microgenomates	Microgenomates bacterium OLB22	ASM156751v1
Bacteria	Nitrospirae-3-1	Nitrospirae	*Leptospirillum ferriphilum*	ASM29923v1
Bacteria	Nitrospirae-3-2	Nitrospirae	*Nitrospira moscoviensis*	ASM127377v1
Bacteria	Nitrospirae-3-3	Nitrospirae	*Nitrospira defluvii*	ASM19681v1

Domain	Code name of species	Phylum	Species	Accession number
Bacteria	Candidatus-Omnitrophica-3-1	Candidatus Omnitrophica	Candidatus Omnitrophica bacterium CG1_02_44_16	ASM187399v1
Bacteria	Candidatus-Omnitrophica-3-2	Candidatus Omnitrophica	Omnitrophica bacterium RIFCSPHIGHO2_02_FULL_46_11	ASM180405v1
Bacteria	Candidatus-Omnitrophica-3-3	Candidatus Omnitrophica	Omnitrophica bacterium RIFCSPLOWO2_01_FULL_50_24	ASM180422v1
Bacteria	Candidatus-Parcubacteria-3-1	Candidatus Parcubacteria	Parcubacteria bacterium DG_74_3	ASM130298v1
Bacteria	Candidatus-Parcubacteria-3-2	Candidatus Parcubacteria	Parcubacteria bacterium SG8_24	ASM130318v1
Bacteria	Candidatus-Parcubacteria-3-3	Candidatus Parcubacteria	Parcubacteria bacterium OLB19	ASM156736v1
Bacteria	Candidatus-Parcubacteria-4	Candidatus Parcubacteria	Parcubacteria bacterium C7867-004	ASM118907v1
Bacteria	Candidatus-Parcubacteria-5	Candidatus Parcubacteria	Parcubacteria bacterium RAAC4_OD1_1	RAAC_OD1_1.0
Bacteria	Candidatus-Parcubacteria-6	Candidatus Parcubacteria	Candidatus Parcubacteria bacterium 4484_255	ASM208514v1
Bacteria	Candidatus-Parcubacteria-7	Candidatus Parcubacteria	Candidatus Parcubacteria bacterium A4	ASM208389v1
Bacteria	Candidatus-Parcubacteria-8	Candidatus Parcubacteria	Parcubacteria bacterium 32_520	ASM150863v1
Bacteria	Candidatus-Parcubacteria-10	Candidatus Parcubacteria	Parcubacteria bacterium 33_209	ASM150830v1
Bacteria	Candidatus-Parcubacteria-11	Candidatus Parcubacteria	Parcubacteria bacterium DG_72	ASM130313v1
Bacteria	Candidatus-Parcubacteria-12	Candidatus Parcubacteria	Parcubacteria bacterium DG_74_1	ASM130312v1
Bacteria	Candidatus-Parcubacteria-14	Candidatus Parcubacteria	Parcubacteria bacterium C7867-008	ASM118921v1
Bacteria	Candidatus-Parcubacteria-15	Candidatus Parcubacteria	Parcubacteria bacterium C7867-006	ASM118915v1
Bacteria	Candidatus-Parcubacteria-16	Candidatus Parcubacteria	Parcubacteria bacterium C7867-005	ASM118916v1
Bacteria	Candidatus-Parcubacteria-18	Candidatus Parcubacteria	Parcubacteria bacterium C7867-002	ASM118910v1
Bacteria	Candidatus-Peregrinibacteria-3-1	Candidatus Peregrinibacteria	Candidatus Peribacteria bacterium GW2011_GWB1_54_5	ASM100473v1
Bacteria	Candidatus-Peregrinibacteria-3-2	Candidatus Peregrinibacteria	Candidatus Peribacteria bacterium GW2011_GWC2_54_8	ASM100477v1
Bacteria	Candidatus-Peregrinibacteria-3-3	Candidatus Peregrinibacteria	Candidatus *Peribacter riflensis*	ASM143075v1
Bacteria	Planctomycetes-3-1	Planctomycetes	*Kuenenia stuttgartiensis*	KustCH1
Bacteria	Planctomycetes-3-2	Planctomycetes	*Phycisphaera mikurensis*	ASM28411v1
Bacteria	Planctomycetes-3-3	Planctomycetes	*Singulisphaera acidiphila*	ASM24245v3
Bacteria	Candidatus-Poribacteria-2-1	Candidatus Poribacteria	Candidatus Poribacteria bacterium WGA-4G	ASM36458v1

Domain	Code name of species	Phylum	Species	Accession number
Bacteria	Candidatus-Poribacteria-2-2	Candidatus Poribacteria	Candidatus Poribacteria bacterium WGA-4E	ASM37228v1
Bacteria	Proteobacteria-6-1	Proteobacteria	*Acidithiobacillus ferrooxidans*	ASM2148v1
Bacteria	Proteobacteria-6-2	Proteobacteria	*Starkeya novella*	ASM9292v1
Bacteria	Proteobacteria-6-3	Proteobacteria	*Thauera humireducens*	ASM105199v2
Bacteria	Proteobacteria-6-4	Proteobacteria	*Geobacter sulfurreducens*	ASM798v2
Bacteria	Proteobacteria-6-5	Proteobacteria	*Escherichia coli*	ASM886v1
Bacteria	Proteobacteria-6-6	Proteobacteria	*Mariprofundus ferrooxydans*	ASM15376v1
Bacteria	Rhodothermaeota-2-1	Rhodothermaeota	*Balneola vulgaris*	ASM37546v1
Bacteria	Rhodothermaeota-2-2	Rhodothermaeota	*Salinibacter ruber*	/
Bacteria	Candidatus-Saccharibacteria-3-1	Candidatus Saccharibacteria	*Saccharimonas aalborgensis*	ASM39243v1
Bacteria	Candidatus-Saccharibacteria-3-2	Candidatus Saccharibacteria	Candidatus Saccharibacteria bacterium RAAC3_TM7_1	ASM50391v1
Bacteria	Candidatus-Saccharibacteria-3-3	Candidatus Saccharibacteria	Candidatus Saccharibacteria oral taxon TM7x	ASM80362v1
Bacteria	Spirochaetes-4-1	Spirochaetes	*Brachyspira pilosicoli*	ASM32566v1
Bacteria	Spirochaetes-4-2	Spirochaetes	*Brevinema andersonii*	IMG-taxon 2597490362 annotated assembly
Bacteria	Spirochaetes-4-3	Spirochaetes	*Leptospira santarosai*	ASM31317v2
Bacteria	Spirochaetes-4-4	Spirochaetes	*Borreliella burgdorferi*	BorBurgIPT112
Bacteria	Synergistetes-3-1	Synergistetes	*Aminomonas paucivorans*	ASM16579v1
Bacteria	Synergistetes-3-2	Synergistetes	*Thermanaerovibrio acidaminovorans*	ASM2490v1
Bacteria	Synergistetes-3-3	Synergistetes	*Aminobacterium colombiense*	ASM2588v1
Bacteria	Tenericutes-3-1	Tenericutes	*Acholeplasma laidlawii*	ASM1878v1
Bacteria	Tenericutes-3-2	Tenericutes	*Mycoplasma testudinis*	ASM68779v1
Bacteria	Tenericutes-3-3	Tenericutes	*Spiroplasma poulsonii*	ASM82052v1
Bacteria	Thermodesulfobacteria-3-1	Thermodesulfobacteria	*Thermosulfurimonas dismutans*	ASM165258v1
Bacteria	Thermodesulfobacteria-3-2	Thermodesulfobacteria	*Thermodesulfobacterium geofontis*	ASM21597v1
Bacteria	Thermodesulfobacteria-3-3	Thermodesulfobacteria	*Thermodesulfobacterium commune*	ASM73401v1

Domain	Code name of species	Phylum	Species	Accession number
Bacteria	Thermotogae-4-1	Thermotogae	*Kosmotoga olearia*	ASM2332v1
Bacteria	Thermotogae-4-2	Thermotogae	*Mesoaciditoga lauensis*	ASM74545v1
Bacteria	Thermotogae-4-3	Thermotogae	*Petrotoga mobilis*	ASM150805v1
Bacteria	Thermotogae-4-4	Thermotogae	*Thermotoga naphthophila*	ASM2510v1
Bacteria	Verrucomicrobia-3-1	Verrucomicrobia	*Coraliomargarita akajimensis*	ASM2590v1
Bacteria	Verrucomicrobia-3-2	Verrucomicrobia	*Terrimicrobium sacchariphilum*	ASM161354v1
Bacteria	Verrucomicrobia-3-3	Verrucomicrobia	*Akkermansia muciniphila*	ASM2022v1
Bacteria	Balneolaeota-3-1	Balneolaeota	*Balneola* sp.	ASM165090v1
Bacteria	Balneolaeota-3-2	Balneolaeota	*Aliifodinibius roseus*	IMG-taxon 2695420936 annotated assembly
Bacteria	Balneolaeota-3-3	Balneolaeota	*Gracilimonas tropica*	ASM37542v1
Bacteria	Calditrichaeota-2-1	Calditrichaeota	*Caldithrix abyssi*	ASM188681v1
Bacteria	Calditrichaeota-2-2	Calditrichaeota	*Caldithrix abyssi*	ASM24181v1
Bacteria	Candidatus-division-CPR2-3-1	candidate division CPR2	candidate division CPR2 bacterium GW2011_GWC2_39_35	ASM99506v1
Bacteria	Candidatus-division-CPR2-3-2	candidate division CPR2	candidate division CPR2 bacterium GW2011_GWD2_39_7	ASM99539v1
Bacteria	Candidatus-division-CPR2-3-3	candidate division CPR2	candidate division CPR2 bacterium GW2011_GWC1_39_9	ASM99556v1
Bacteria	Candidatus-division-KD3-62-1-1	candidate division KD3-62	candidate division KD3-62 bacterium DG_56	ASM130280v1
Bacteria	Candidatus-division-NC10-3-1	candidate division NC10	Candidatus *Methylomirabilis oxyfera*	ASM9116v1
Bacteria	Candidatus-division-NC10-3-2	candidate division NC10	candidate division NC10 bacterium RIFCSPLOWO2_02_FULL_66_22	ASM177124v1
Bacteria	Candidatus-division-NC10-3-3	candidate division NC10	candidate division NC10 bacterium RIFCSPLOWO2_12_FULL_66_18	ASM177128v1
Bacteria	Candidatus-division-WWE3-3-1	candidate division WWE3	candidate division WWE3 bacterium RBG_19FT_COMBO_34_6	ASM177257v1
Bacteria	Candidatus-division-WWE3-3-2	candidate division WWE3	candidate division WWE3 bacterium RIFOXYA2_FULL_43_12	ASM177279v1
Bacteria	Candidatus-division-WWE3-3-3	candidate division WWE3	candidate division WWE3 bacterium RBG_19FT_COMBO_53_11	ASM177280v1
Bacteria	Candidatus-Abawacabacteria-1-1	Candidatus Abawacabacteria	Candidatus Abawacabacteria bacterium RBG_16_42_10	ASM177345v1
Bacteria	Candidatus-Amesbacteria-3-1	Candidatus Amesbacteria	Candidatus Amesbacteria bacterium GW2011_GWA1_47_16	ASM100100v1
Bacteria	Candidatus-Amesbacteria-3-2	Candidatus Amesbacteria	Candidatus Amesbacteria bacterium GW2011_GWB1_48_13	ASM100358v1

Domain	Code name of species	Phylum	Species	Accession number
Bacteria	Candidatus-Amesbacteria-3-3	Candidatus Amesbacteria	Candidatus Amesbacteria bacterium RIFCSPHIGHO2_01_FULL_48_32	ASM177361v1
Bacteria	Candidatus-Andersenbacteria-2-1	Candidatus Andersenbacteria	Candidatus Andersenbacteria bacterium RIFCSPHIGHO2_02_FULL_46_16	ASM181706v1
Bacteria	Candidatus-Andersenbacteria-2-2	Candidatus Andersenbacteria	Candidatus Andersenbacteria bacterium RIFCSPHIGHO2_12_FULL_46_9	ASM181713v1
Bacteria	Candidatus-Azambacteria-3-1	Candidatus Azambacteria	Candidatus Azambacteria bacterium GW2011_GWE1_42_9	ASM99579v1
Bacteria	Candidatus-Azambacteria-3-2	Candidatus Azambacteria	Candidatus Azambacteria bacterium GW2011_GWF1_41_10	ASM99631v1
Bacteria	Candidatus-Azambacteria-3-3	Candidatus Azambacteria	Candidatus Azambacteria bacterium RIFCSPHIGHO2_02_FULL_45_18	ASM177405v1
Bacteria	Candidatus-Beckwithbacteria-3-1	Candidatus Beckwithbacteria	Candidatus Beckwithbacteria bacterium RIFCSPLOWO2_02_FULL_49_12	ASM177433v1
Bacteria	Candidatus-Beckwithbacteria-3-2	Candidatus Beckwithbacteria	Candidatus Beckwithbacteria bacterium RBG_13_42_9	ASM177604v1
Bacteria	Candidatus-Beckwithbacteria-3-3	Candidatus Beckwithbacteria	Candidatus Beckwithbacteria bacterium RIFCSPHIGHO2_02_FULL_49_13	ASM177607v1
Bacteria	Candidatus-Blackburnbacteria-3-1	Candidatus Blackburnbacteria	Candidatus Blackburnbacteria bacterium RIFCSPHIGHO2_02_FULL_44_20	ASM181642v1
Bacteria	Candidatus-Blackburnbacteria-3-2	Candidatus Blackburnbacteria	Candidatus Blackburnbacteria bacterium RIFCSPHIGHO2_12_FULL_44_25	ASM181645v1
Bacteria	Candidatus-Blackburnbacteria-3-3	Candidatus Blackburnbacteria	Candidatus Blackburnbacteria bacterium RIFCSPLOWO2_01_FULL_40_20	ASM181647v1
Bacteria	Candidatus-Brennerbacteria-1-1	Candidatus Brennerbacteria	Candidatus Brennerbacteria bacterium RIFOXYC1_FULL_41_11	ASM181721v1
Bacteria	Candidatus-Buchananbacteria-3-1	Candidatus Buchananbacteria	Candidatus Buchananbacteria bacterium RBG_13_39_9	ASM181726v1
Bacteria	Candidatus-Buchananbacteria-3-2	Candidatus Buchananbacteria	Candidatus Buchananbacteria bacterium RIFCSPHIGHO2_02_FULL_56_16	ASM181749v1
Bacteria	Candidatus-Buchananbacteria-3-3	Candidatus Buchananbacteria	Candidatus Buchananbacteria bacterium RIFCSPLOWO2_01_FULL_46_12	ASM181757v1
Bacteria	Candidatus-Campbellbacteria-3-1	Candidatus Campbellbacteria	Candidatus Campbellbacteria bacterium RIFOXYC2_FULL_35_25	ASM177449v1
Bacteria	Candidatus-Campbellbacteria-3-2	Candidatus Campbellbacteria	Candidatus Campbellbacteria bacterium GW2011_GWC1_35_31	ASM99074v1
Bacteria	Candidatus-Campbellbacteria-3-3	Candidatus Campbellbacteria	Candidatus Campbellbacteria bacterium GW2011_GWC2_35_28	ASM99075v1
Bacteria	Candidatus-Chisholmbacteria-3-1	Candidatus Chisholmbacteria	Candidatus Chisholmbacteria bacterium RIFCSPHIGHO2_01_FULL_52_32	ASM181658v1
Bacteria	Candidatus-Chisholmbacteria-3-2	Candidatus Chisholmbacteria	Candidatus Chisholmbacteria bacterium RIFCSPLOWO2_01_FULL_49_14	ASM181691v1
Bacteria	Candidatus-Chisholmbacteria-3-3	Candidatus Chisholmbacteria	Candidatus Chisholmbacteria bacterium RIFCSPLOWO2_01_FULL_50_28	ASM181695v1
Bacteria	Candidatus-Cloacimonetes-3-1	Candidatus Cloacimonetes	Candidatus *Cloacimonas acidaminovorans*	ASM14606v1
Bacteria	Candidatus-Cloacimonetes-3-2	Candidatus Cloacimonetes	Cloacimonetes bacterium JGI 0000039-P09	ASM38080v1
Bacteria	Candidatus-Cloacimonetes-3-3	Candidatus Cloacimonetes	Cloacimonetes bacterium JGI 0000039-I07	ASM38475v1

Domain	Code name of species	Phylum	Species	Accession number
Bacteria	Candidatus-Collierbacteria-3-1	Candidatus Collierbacteria	Candidatus Collierbacteria bacterium RIFOXYB2_FULL_46_14	ASM177463v1
Bacteria	Candidatus-Collierbacteria-3-2	Candidatus Collierbacteria	Candidatus Collierbacteria bacterium RIFOXYC2_FULL_43_15	ASM177467v1
Bacteria	Candidatus-Collierbacteria-3-3	Candidatus Collierbacteria	Candidatus Collierbacteria bacterium RIFOXYD2_FULL_45_13	ASM177468v1
Bacteria	Candidatus-Curtissbacteria-3-1	Candidatus Curtissbacteria	Candidatus Curtissbacteria bacterium GW2011_GWA1_40_16	ASM99599v1
Bacteria	Candidatus-Curtissbacteria-3-2	Candidatus Curtissbacteria	Candidatus Curtissbacteria bacterium RIFCSPLOWO2_02_FULL_40_11	ASM177650v1
Bacteria	Candidatus-Curtissbacteria-3-3	Candidatus Curtissbacteria	Candidatus Curtissbacteria bacterium RIFCSPLOWO2_02_41_11	ASM177505v1
Bacteria	Candidatus-Colwellbacteria-3-1	Candidatus Colwellbacteria	Candidatus Colwellbacteria bacterium RIFCSPHIGHO2_02_FULL_43_15	ASM181767v1
Bacteria	Candidatus-Colwellbacteria-3-2	Candidatus Colwellbacteria	Candidatus Colwellbacteria bacterium RIFCSPHIGHO2_12_FULL_43_12	ASM181771v1
Bacteria	Candidatus-Colwellbacteria-3-3	Candidatus Colwellbacteria	Candidatus Colwellbacteria bacterium RIFCSPLOWO2_12_FULL_43_11	ASM181780v1
Bacteria	Candidatus-Dadabacteria-1-1	Candidatus Dadabacteria	Candidatus Dadabacteria bacterium CSP1-2	ASM144344v1
Bacteria	Candidatus-Daviesbacteria-3-1	Candidatus Daviesbacteria	Candidatus Daviesbacteria bacterium GW2011_GWA2_38_24	ASM99264v1
Bacteria	Candidatus-Daviesbacteria-3-2	Candidatus Daviesbacteria	Candidatus Daviesbacteria bacterium RIFCSPLOWO2_02_FULL_38_18	ASM177743v1
Bacteria	Candidatus-Daviesbacteria-3-3	Candidatus Daviesbacteria	Candidatus Daviesbacteria bacterium RIFCSPHIGHO2_02_FULL_39_41	ASM177706v1
Bacteria	Candidatus-Doudnabacteria-3-1	Candidatus Doudnabacteria	Candidatus Doudnabacteria bacterium RIFCSPHIGHO2_01_43_10	ASM177754v1
Bacteria	Candidatus-Doudnabacteria-3-2	Candidatus Doudnabacteria	Candidatus Doudnabacteria bacterium RIFCSPHIGHO2_02_FULL_42_25	ASM177766v1
Bacteria	Candidatus-Doudnabacteria-3-3	Candidatus Doudnabacteria	Candidatus Doudnabacteria bacterium RIFCSPHIGHO2_12_FULL_42_22	ASM177998v1
Bacteria	Candidatus-Edwardsbacteria-3-1	Candidatus Edwardsbacteria	Candidatus Edwardsbacteria bacterium RIFOXYD12_FULL_50_11	ASM177794v1
Bacteria	Candidatus-Edwardsbacteria-3-2	Candidatus Edwardsbacteria	Candidatus Edwardsbacteria bacterium GWF2_54_11	ASM177789v1
Bacteria	Candidatus-Edwardsbacteria-3-3	Candidatus Edwardsbacteria	Candidatus Edwardsbacteria bacterium RifOxyA12_full_54_48	ASM177791v1
Bacteria	Candidatus-Eisenbacteria-1-1	Candidatus Eisenbacteria	Candidatus Eisenbacteria bacterium RBG_16_71_46	ASM178016v1
Bacteria	Candidatus-Falkowbacteria-3-1	Candidatus Falkowbacteria	Candidatus Falkowbacteria bacterium GW2011_GWF2_38_1205	ASM99237v1
Bacteria	Candidatus-Falkowbacteria-3-2	Candidatus Falkowbacteria	Candidatus Falkowbacteria bacterium GW2011_GWC2_38_22	ASM99257v2
Bacteria	Candidatus-Falkowbacteria-3-3	Candidatus Falkowbacteria	Candidatus Falkowbacteria bacterium GW2011_GWF1_38_22	ASM99261v1
Bacteria	Candidatus-Fischerbacteria-1-1	Candidatus Fischerbacteria	Candidatus Fischerbacteria bacterium RBG_13_37_8	ASM178038v1
Bacteria	Candidatus-Giovannonibacteria-3-1	Candidatus Giovannonibacteria	Candidatus Giovannonibacteria bacterium GW2011_GWB1_43_13	ASM99908v1

Domain	Code name of species	Phylum	Species	Accession number
Bacteria	Candidatus-Giovannonibacteria-3-2	Candidatus Giovannonibacteria	Candidatus Giovannonibacteria bacterium RIFCSPHIGHO2_01_45_12	ASM177841v1
Bacteria	Candidatus-Giovannonibacteria-3-3	Candidatus Giovannonibacteria	Candidatus Giovannonibacteria bacterium RIFCSPHIGHO2_02_43_13	ASM177855v1
Bacteria	Candidatus-Gottesmanbacteria-3-1	Candidatus Gottesmanbacteria	Candidatus Gottesmanbacteria bacterium RBG_16_52_11	ASM177909v1
Bacteria	Candidatus-Gottesmanbacteria-3-2	Candidatus Gottesmanbacteria	Candidatus Gottesmanbacteria bacterium RIFCSPHIGHO2_01_FULL_40_15	ASM177911v1
Bacteria	Candidatus-Gottesmanbacteria-3-3	Candidatus Gottesmanbacteria	Candidatus Gottesmanbacteria bacterium RIFCSPHIGHO2_01_FULL_42_27	ASM177913v1
Bacteria	Candidatus-Harrisonbacteria-3-1	Candidatus Harrisonbacteria	Candidatus Harrisonbacteria bacterium RIFCSPLOWO2_01_FULL_44_18	ASM181791v1
Bacteria	Candidatus-Harrisonbacteria-3-2	Candidatus Harrisonbacteria	Candidatus Harrisonbacteria bacterium RIFCSPLOWO2_02_FULL_41_13b	ASM181799v1
Bacteria	Candidatus-Harrisonbacteria-3-3	Candidatus Harrisonbacteria	Candidatus Harrisonbacteria bacterium RIFCSPHIGHO2_02_FULL_40_20	ASM181867v1
Bacteria	Candidatus-Jacksonbacteria-3-1	Candidatus Jacksonbacteria	Candidatus Jacksonbacteria bacterium RIFCSPHIGHO2_02_FULL_44_25	ASM181807v1
Bacteria	Candidatus-Jacksonbacteria-3-2	Candidatus Jacksonbacteria	Candidatus Jacksonbacteria bacterium RIFCSPLOWO2_02_FULL_44_20	ASM181815v1
Bacteria	Candidatus-Jacksonbacteria-3-3	Candidatus Jacksonbacteria	Candidatus Jacksonbacteria bacterium RIFCSPLOWO2_12_FULL_44_15b	ASM181817v1
Bacteria	Candidatus-Jorgensenbacteria-3-1	Candidatus Jorgensenbacteria	Candidatus Jorgensenbacteria bacterium GW2011_GWA1_48_13	ASM100352v1
Bacteria	Candidatus-Jorgensenbacteria-3-2	Candidatus Jorgensenbacteria	Candidatus Jorgensenbacteria bacterium GW2011_GWB1_50_10	ASM100410v1
Bacteria	Candidatus-Jorgensenbacteria-3-3	Candidatus Jorgensenbacteria	Candidatus Jorgensenbacteria bacterium GWA1_54_12	ASM177950v1
Bacteria	Candidatus-Kaiserbacteria-3-1	Candidatus Kaiserbacteria	Candidatus Kaiserbacteria bacterium RIFCSPLOWO2_12_FULL_45_26	ASM178180v1
Bacteria	Candidatus-Kaiserbacteria-3-2	Candidatus Kaiserbacteria	Candidatus Kaiserbacteria bacterium RIFCSPLOWO2_02_FULL_45_11b	ASM178343v1
Bacteria	Candidatus-Kaiserbacteria-3-3	Candidatus Kaiserbacteria	Candidatus Kaiserbacteria bacterium RIFCSPLOWO2_12_FULL_50_10	ASM178351v1
Bacteria	Candidatus-Kerfeldbacteria-3-1	Candidatus Kerfeldbacteria	Candidatus Kerfeldbacteria bacterium RIFCSPLOWO2_02_FULL_42_19	ASM181831v1
Bacteria	Candidatus-Kerfeldbacteria-3-2	Candidatus Kerfeldbacteria	Candidatus Kerfeldbacteria bacterium RIFCSPLOWO2_12_FULL_43_9	ASM181833v1
Bacteria	Candidatus-Kerfeldbacteria-3-3	Candidatus Kerfeldbacteria	Candidatus Kerfeldbacteria bacterium RIFCSPHIGHO2_02_FULL_42_14	ASM181873v1
Bacteria	Candidatus-Komeilibacteria-3-1	Candidatus Komeilibacteria	Candidatus Komeilibacteria bacterium RIFCSPHIGHO2_01_FULL_52_14	ASM181836v1
Bacteria	Candidatus-Komeilibacteria-3-2	Candidatus Komeilibacteria	Candidatus Komeilibacteria bacterium RIFOXYC1_FULL_37_11	ASM181844v1
Bacteria	Candidatus-Komeilibacteria-3-3	Candidatus Komeilibacteria	Candidatus Komeilibacteria bacterium RIFOXYC2_FULL_45_12	ASM181847v1
Bacteria	Candidatus-Kryptonia-3-1	Candidatus Kryptonia	*Candidatus Kryptobacter tengchongensis*	JGI assembly
Bacteria	Candidatus-Kryptonia-3-2	Candidatus Kryptonia	*Candidatus Kryptobacter tengchongensis*	JGI assembly

Domain	Code name of species	Phylum	Species	Accession number
Bacteria	Candidatus-Kryptonia-3-3	Candidatus Kryptonia	*Candidatus Kryptonium thompsoni*	JGI assembly
Bacteria	Candidatus-Kuenenbacteria-3-1	Candidatus Kuenenbacteria	Candidatus Kuenenbacteria bacterium RIFCSPLOWO2_12_FULL_42_13	ASM178364v1
Bacteria	Candidatus-Kuenenbacteria-3-2	Candidatus Kuenenbacteria	Candidatus Kuenenbacteria bacterium CG1_02_38_13	ASM187136v1
Bacteria	Candidatus-Kuenenbacteria-3-3	Candidatus Kuenenbacteria	Candidatus Kuenenbacteria bacterium RIFCSPLOWO2_02_FULL_42_16	ASM178193v1
Bacteria	Candidatus-Levybacteria-3-1	Candidatus Levybacteria	Candidatus Levybacteria bacterium RIFCSPLOWO2_02_FULL_37_18	ASM178411v1
Bacteria	Candidatus-Levybacteria-3-2	Candidatus Levybacteria	Candidatus Levybacteria bacterium RIFCSPLOWO2_01_FULL_37_20	ASM178244v1
Bacteria	Candidatus-Levybacteria-3-3	Candidatus Levybacteria	Candidatus Levybacteria bacterium RIFCSPLOWO2_01_FULL_39_24	ASM178252v1
Bacteria	Candidatus-Lindowbacteria-2-1	Candidatus Lindowbacteria	Candidatus Lindowbacteria bacterium RIFCSPLOWO2_02_FULL_62_12	ASM178279v1
Bacteria	Candidatus-Lindowbacteria-2-2	Candidatus Lindowbacteria	Candidatus Lindowbacteria bacterium RIFCSPLOWO2_12_FULL_62_27	ASM178417v1
Bacteria	Candidatus-Liptonbacteria-3-1	Candidatus Liptonbacteria	Candidatus Liptonbacteria bacterium RIFCSPHIGHO2_12_FULL_60_13	ASM181859v1
Bacteria	Candidatus-Liptonbacteria-3-2	Candidatus Liptonbacteria	Candidatus Liptonbacteria bacterium RIFCSPLOWO2_01_FULL_52_25	ASM181862v1
Bacteria	Candidatus-Liptonbacteria-3-3	Candidatus Liptonbacteria	Candidatus Liptonbacteria bacterium RIFCSPLOWO2_01_FULL_56_20	ASM181865v1
Bacteria	Candidatus-Llyodbacteria-3-1	Candidatus Llyodbacteria	Candidatus Lloydbacteria bacterium RIFCSPLOWO2_02_FULL_51_11	ASM181907v1
Bacteria	Candidatus-Llyodbacteria-3-2	Candidatus Llyodbacteria	Candidatus Lloydbacteria bacterium RIFCSPLOWO2_02_FULL_54_12	ASM181909v1
Bacteria	Candidatus-Llyodbacteria-3-3	Candidatus Llyodbacteria	Candidatus Lloydbacteria bacterium RIFCSPHIGHO2_02_FULL_50_18	ASM182139v1
Bacteria	Candidatus-Magasanikbacteria-3-1	Candidatus Magasanikbacteria	Candidatus Magasanikbacteria bacterium RIFOXYD1_FULL_40_23	ASM178321v1
Bacteria	Candidatus-Magasanikbacteria-3-2	Candidatus Magasanikbacteria	Candidatus Magasanikbacteria bacterium GW2011_GWC2_42_27	ASM99863v1
Bacteria	Candidatus-Magasanikbacteria-3-3	Candidatus Magasanikbacteria	Candidatus Magasanikbacteria bacterium RIFOXYC2_FULL_42_28	ASM178443v1
Bacteria	Candidatus-Moranbacteria-3-1	Candidatus Moranbacteria	Candidatus Moranbacteria bacterium GW2011_GWF1_44_4	ASM99976v1
Bacteria	Candidatus-Moranbacteria-3-2	Candidatus Moranbacteria	Candidatus Moranbacteria bacterium GW2011_GWC1_45_18	ASM100152v1
Bacteria	Candidatus-Moranbacteria-3-3	Candidatus Moranbacteria	Candidatus Moranbacteria bacterium GW2011_GWE1_49_15	ASM100385v1
Bacteria	Candidatus-Nealsonbacteria-3-1	Candidatus Nealsonbacteria	Candidatus Nealsonbacteria bacterium RBG_13_42_11	ASM182145v1
Bacteria	Candidatus-Nealsonbacteria-3-2	Candidatus Nealsonbacteria	Candidatus Nealsonbacteria bacterium RIFOXYC1_FULL_40_7	ASM182157v1
Bacteria	Candidatus-Nealsonbacteria-3-3	Candidatus Nealsonbacteria	Candidatus Nealsonbacteria bacterium RIFOXYD1_FULL_39_11	ASM182158v1
Bacteria	Candidatus-Niyogibacteria-3-1	Candidatus Niyogibacteria	Candidatus Niyogibacteria bacterium RIFCSPLOWO2_01_FULL_45_48	ASM181939v1

Domain	Code name of species	Phylum	Species	Accession number
Bacteria	Candidatus-Niyogibacteria-3-2	Candidatus Niyogibacteria	Candidatus Niyogibacteria bacterium RIFCSPLOWO2_02_FULL_45_13	ASM181941v1
Bacteria	Candidatus-Niyogibacteria-3-3	Candidatus Niyogibacteria	Candidatus Niyogibacteria bacterium RIFCSPLOWO2_12_FULL_41_13	ASM182161v1
Bacteria	Candidatus-Nomurabacteria-3-1	Candidatus Nomurabacteria	Candidatus Nomurabacteria bacterium RIFOXYB1_FULL_36_10	ASM178625v1
Bacteria	Candidatus-Nomurabacteria-3-2	Candidatus Nomurabacteria	Candidatus Nomurabacteria bacterium GW2011_GWD2_36_14	ASM99140v1
Bacteria	Candidatus-Nomurabacteria-3-3	Candidatus Nomurabacteria	Candidatus Nomurabacteria bacterium CG2_30_43_9	ASM187330v1
Bacteria	Candidatus-Pacebacteria-3-1	Candidatus Pacebacteria	Candidatus Pacebacteria bacterium GW2011_GWF1_36_5	ASM99172v1
Bacteria	Candidatus-Pacebacteria-3-2	Candidatus Pacebacteria	Candidatus Pacebacteria bacterium GW2011_GWF2_38_9	ASM99318v1
Bacteria	Candidatus-Pacebacteria-3-3	Candidatus Pacebacteria	Candidatus Pacebacteria bacterium RIFOXYA1_FULL_38_18	ASM178740v1
Bacteria	Candidatus-Portnoybacteria-3-1	Candidatus Portnoybacteria	Candidatus Portnoybacteria bacterium RBG_19FT_COMBO_36_7	ASM181949v1
Bacteria	Candidatus-Portnoybacteria-3-2	Candidatus Portnoybacteria	Candidatus Portnoybacteria bacterium RIFCSPHIGHO2_01_FULL_40_12b	ASM181953v1
Bacteria	Candidatus-Portnoybacteria-3-3	Candidatus Portnoybacteria	Candidatus Portnoybacteria bacterium RIFCSPLOWO2_01_FULL_38_39	ASM181958v1
Bacteria	Candidatus-Raymondbacteria-3-1	Candidatus Raymondbacteria	Candidatus Raymondbacteria bacterium RIFOXYC2_FULL_50_21	ASM178783v1
Bacteria	Candidatus-Raymondbacteria-3-2	Candidatus Raymondbacteria	Candidatus Raymondbacteria bacterium RifOxyA12_full_50_37	ASM178919v1
Bacteria	Candidatus-Raymondbacteria-3-3	Candidatus Raymondbacteria	Candidatus Raymondbacteria bacterium RIFOXYA2_FULL_49_16	ASM178920v1
Bacteria	Candidatus-Roizmanbacteria-3-1	Candidatus Roizmanbacteria	Candidatus Roizmanbacteria bacterium GW2011_GWC1_37_12	ASM99113v1
Bacteria	Candidatus-Roizmanbacteria-3-2	Candidatus Roizmanbacteria	Candidatus Roizmanbacteria bacterium RIFOXYC2_FULL_38_9	ASM178830v1
Bacteria	Candidatus-Roizmanbacteria-3-3	Candidatus Roizmanbacteria	Candidatus Roizmanbacteria bacterium RIFCSPHIGHO2_12_41_18	ASM178953v1
Bacteria	Candidatus-Ryanbacteria-3-1	Candidatus Ryanbacteria	Candidatus Ryanbacteria bacterium RIFCSPHIGHO2_02_FULL_47_25	ASM181985v1
Bacteria	Candidatus-Ryanbacteria-3-2	Candidatus Ryanbacteria	Candidatus Ryanbacteria bacterium RIFCSPHIGHO2_02_FULL_48_12	ASM181987v1
Bacteria	Candidatus-Ryanbacteria-3-3	Candidatus Ryanbacteria	Candidatus Ryanbacteria bacterium RIFCSPHIGHO2_12_FULL_47_12b	ASM181990v1
Bacteria	Candidatus-Schekmanbacteria-1-1	Candidatus Schekmanbacteria	Candidatus Schekmanbacteria bacterium RIFCSPLOWO2_02_FULL_38_14	ASM179085v1
Bacteria	Candidatus-Shapirobacteria-3-1	Candidatus Shapirobacteria	Candidatus Shapirobacteria bacterium GW2011_GWF1_38_23	ASM99260v1
Bacteria	Candidatus-Shapirobacteria-3-2	Candidatus Shapirobacteria	Candidatus Shapirobacteria bacterium GW2011_GWE2_38_30	ASM99272v1
Bacteria	Candidatus-Shapirobacteria-3-3	Candidatus Shapirobacteria	Candidatus Shapirobacteria bacterium RIFOXYD1_FULL_38_32	ASM179090v1
Bacteria	Candidatus-Spechtbacteria-3-1	Candidatus Spechtbacteria	Candidatus Spechtbacteria bacterium RIFCSPHIGHO2_02_FULL_43_15b	ASM181998v1

Domain	Code name of species	Phylum	Species	Accession number
Bacteria	Candidatus-Spechtbacteria-3-2	Candidatus Spechtbacteria	Candidatus Spechtbacteria bacterium RIFCSPLOWO2_12_FULL_38_22	ASM182005v1
Bacteria	Candidatus-Spechtbacteria-3-3	Candidatus Spechtbacteria	Candidatus Spechtbacteria bacterium RIFCSPHIGHO2_12_FULL_38_30	ASM182183v1
Bacteria	Candidatus-Staskawiczbacteria-3-1	Candidatus Staskawiczbacteria	Candidatus Staskawiczbacteria bacterium RIFOXYA1_FULL_37_15	ASM182029v1
Bacteria	Candidatus-Staskawiczbacteria-3-2	Candidatus Staskawiczbacteria	Candidatus Staskawiczbacteria bacterium RIFOXYA12_FULL_37_10	ASM182032v1
Bacteria	Candidatus-Staskawiczbacteria-3-3	Candidatus Staskawiczbacteria	Candidatus Staskawiczbacteria bacterium RIFOXYA2_FULL_32_7	ASM182035v1
Bacteria	Candidatus-Sungbacteria-3-1	Candidatus Sungbacteria	Candidatus Sungbacteria bacterium RIFCSPLOWO2_01_FULL_59_16	ASM182238v1
Bacteria	Candidatus-Sungbacteria-3-2	Candidatus Sungbacteria	Candidatus Sungbacteria bacterium RIFCSPLOWO2_02_FULL_54_10	ASM182245v1
Bacteria	Candidatus-Sungbacteria-3-3	Candidatus Sungbacteria	Candidatus Sungbacteria bacterium RIFCSPLOWO2_01_FULL_47_32	ASM182068v1
Bacteria	Candidatus-Taylorbacteria-3-1	Candidatus Taylorbacteria	Candidatus Taylorbacteria bacterium RIFCSPHIGHO2_02_FULL_45_35	ASM182098v1
Bacteria	Candidatus-Taylorbacteria-3-2	Candidatus Taylorbacteria	Candidatus Taylorbacteria bacterium RIFCSPLOWO2_02_FULL_45_10b	ASM182123v1
Bacteria	Candidatus-Taylorbacteria-3-3	Candidatus Taylorbacteria	Candidatus Taylorbacteria bacterium RIFCSPLOWO2_01_FULL_45_34b	ASM182267v1
Bacteria	Candidatus-Terrybacteria-3-1	Candidatus Terrybacteria	Candidatus Terrybacteria bacterium RIFCSPHIGHO2_01_FULL_43_35	ASM182281v1
Bacteria	Candidatus-Terrybacteria-3-2	Candidatus Terrybacteria	Candidatus Terrybacteria bacterium RIFCSPHIGHO2_02_FULL_43_14	ASM182286v1
Bacteria	Candidatus-Terrybacteria-3-3	Candidatus Terrybacteria	Candidatus Terrybacteria bacterium RIFCSPLOWO2_01_FULL_40_23	ASM182289v1
Bacteria	Candidatus-Uhrbacteria-3-1	Candidatus Uhrbacteria	Candidatus Uhrbacteria bacterium GW2011_GWF2_46_218	ASM100256v1
Bacteria	Candidatus-Uhrbacteria-3-2	Candidatus Uhrbacteria	Candidatus Uhrbacteria bacterium RIFCSPLOWO2_01_FULL_47_17	ASM179130v1
Bacteria	Candidatus-Uhrbacteria-3-3	Candidatus Uhrbacteria	Candidatus Uhrbacteria bacterium RIFOXYB12_FULL_58_10	ASM179164v1
Bacteria	Candidatus-Veblenbacteria-3-1	Candidatus Veblenbacteria	Candidatus Veblenbacteria bacterium RIFOXYC2_FULL_42_11	ASM182298v1
Bacteria	Candidatus-Veblenbacteria-3-2	Candidatus Veblenbacteria	Candidatus Veblenbacteria bacterium RIFOXYD1_FULL_43_11	ASM182301v1
Bacteria	Candidatus-Veblenbacteria-3-3	Candidatus Veblenbacteria	Candidatus Veblenbacteria bacterium RIFOXYA2_FULL_43_9	ASM182475v1
Bacteria	Candidatus-Vogelbacteria-3-1	Candidatus Vogelbacteria	Candidatus Vogelbacteria bacterium GWA1_51_14	ASM182302v1
Bacteria	Candidatus-Vogelbacteria-3-2	Candidatus Vogelbacteria	Candidatus Vogelbacteria bacterium RIFOXYB1_FULL_42_16	ASM182305v1
Bacteria	Candidatus-Vogelbacteria-3-3	Candidatus Vogelbacteria	Candidatus Vogelbacteria bacterium RIFOXYD1_FULL_51_18	ASM182309v1
Bacteria	Candidatus-Wildermuthbacteria-3-1	Candidatus Wildermuthbacteria	Candidatus Wildermuthbacteria bacterium RIFCSPLOWO2_01_FULL_48_35	ASM182337v1
Bacteria	Candidatus-Wildermuthbacteria-3-2	Candidatus Wildermuthbacteria	Candidatus Wildermuthbacteria bacterium RIFCSPLOWO2_02_FULL_47_10	ASM182341v1

Domain	Code name of species	Phylum	Species	Accession number
Bacteria	Candidatus-Wildermuthbacteria-3-3	Candidatus Wildermuthbacteria	Candidatus Wildermuthbacteria bacterium RIFCSPHIGHO2_01_FULL_47_27	ASM182488v1
Bacteria	Candidatus-Woesebacteria-3-1	Candidatus Woesebacteria	Candidatus Woesebacteria bacterium GW2011_GWB1_39_12	ASM99340v1
Bacteria	Candidatus-Woesebacteria-3-2	Candidatus Woesebacteria	Candidatus Woesebacteria bacterium RIFCSPHIGHO2_01_FULL_40_22	ASM179237v1
Bacteria	Candidatus-Woesebacteria-3-3	Candidatus Woesebacteria	Candidatus Woesebacteria bacterium RIFOXYA1_FULL_38_9	ASM179333v1
Bacteria	Candidatus-Wolfebacteria-3-1	Candidatus Wolfebacteria	Candidatus Wolfebacteria bacterium GW2011_GWD2_47_17	ASM99847v1
Bacteria	Candidatus-Wolfebacteria-3-2	Candidatus Wolfebacteria	Candidatus Wolfebacteria bacterium RIFCSPLOWO2_01_FULL_47_17b	ASM179386v1
Bacteria	Candidatus-Wolfebacteria-3-3	Candidatus Wolfebacteria	Candidatus Wolfebacteria bacterium RIFOXYB1_FULL_54_12	ASM179389v1
Bacteria	Candidatus-Woykebacteria-3-1	Candidatus Woykebacteria	Candidatus Woykebacteria bacterium RIFCSPHIGHO2_02_FULL_43_16b	ASM181679v1
Bacteria	Candidatus-Woykebacteria-3-2	Candidatus Woykebacteria	Candidatus Woykebacteria bacterium RIFCSPHIGHO2_12_FULL_43_10	ASM181681v1
Bacteria	Candidatus-Woykebacteria-3-3	Candidatus Woykebacteria	Candidatus Woykebacteria bacterium RIFCSPLOWO2_01_FULL_43_14	ASM181683v1
Bacteria	Candidatus-Yanofskybacteria-3-1	Candidatus Yanofskybacteria	Candidatus Yanofskybacteria bacterium RIFCSPHIGHO2_12_FULL_45_19b	ASM179460v1
Bacteria	Candidatus-Yanofskybacteria-3-2	Candidatus Yanofskybacteria	Candidatus Yanofskybacteria bacterium RIFCSPLOWO2_01_FULL_39_28	ASM179463v1
Bacteria	Candidatus-Yanofskybacteria-3-3	Candidatus Yanofskybacteria	Candidatus Yanofskybacteria bacterium RIFCSPLOWO2_02_FULL_44_18	ASM179475v1
Bacteria	Candidatus-Yonathbacteria-3-1	Candidatus Yonathbacteria	Candidatus Yonathbacteria bacterium RIFCSPHIGHO2_01_FULL_44_41	ASM182349v1
Bacteria	Candidatus-Yonathbacteria-3-2	Candidatus Yonathbacteria	Candidatus Yonathbacteria bacterium RIFOXYC1_FULL_52_10	ASM182359v1
Bacteria	Candidatus-Yonathbacteria-3-3	Candidatus Yonathbacteria	Candidatus Yonathbacteria bacterium RIFOXYD1_FULL_52_36	ASM182360v1
Bacteria	Candidatus-Zambryskibacteria-3-1	Candidatus Zambryskibacteria	Candidatus Zambryskibacteria bacterium RIFCSPHIGHO2_12_FULL_38_37	ASM182381v1
Bacteria	Candidatus-Zambryskibacteria-3-2	Candidatus Zambryskibacteria	Candidatus Zambryskibacteria bacterium RIFCSPLOWO2_02_FULL_51_21	ASM182425v1
Bacteria	Candidatus-Zambryskibacteria-3-3	Candidatus Zambryskibacteria	Candidatus Zambryskibacteria bacterium RIFOXYC1_FULL_39_10	ASM182427v1
Bacteria	Ignavibacteriae-3-1	Ignavibacteriae	*Melioribacter* sp.	ASM180334v1
Bacteria	Ignavibacteriae-3-2	Ignavibacteriae	*Ignavibacterium album*	ASM25840v1
Bacteria	Ignavibacteriae-3-3	Ignavibacteriae	*Melioribacter roseus*	ASM27914v1
Bacteria	Kiritimatiellaeota-1-1	Kiritimatiellaeota	*Kiritimatiella glycovorans*	ASM101765v1
Bacteria	Candidatus-division-CPR3-3-1	candidate division CPR3	candidate division CPR3 bacterium GW2011_GWF2_35_18	ASM99057v1
Bacteria	Candidatus-division-CPR3-3-2	candidate division CPR3	candidate division CPR3 bacterium GWF2_35_18	ASM177113v1

Domain	Code name of species	Phylum	Species	Accession number
Bacteria	Candidatus-division-CPR3-3-3	candidate division CPR3	candidate division CPR3 bacterium RIFOXYA2_FULL_35_13	ASM177115v1
Bacteria	Candidatus-Fermentibacteria-3-1	Candidatus Fermentibacteria	candidate division Hyd24-12 bacterium Ran_1	ASM161860v1
Bacteria	Candidatus-Fermentibacteria-3-2	Candidatus Fermentibacteria	candidate division Hyd24-12 bacterium Dam_1	ASM161861v1
Bacteria	Candidatus-Fermentibacteria-3-3	Candidatus Fermentibacteria	candidate division Hyd24-12 bacterium Vib_1	ASM161862v1
Bacteria	Candidatus-division-WOR-3-1-1	candidate division WOR-3	candidate division WOR-3 bacterium RBG_13_43_14	ASM177173v1
Bacteria	Candidatus-division-Zixibacteria-3-1	candidate division Zixibacteria	candidate division Zixibacteria bacterium RBG-1	RBG_1
Bacteria	Candidatus-division-Zixibacteria-3-2	candidate division Zixibacteria	candidate division Zixibacteria bacterium SM1_73	ASM130422v1
Bacteria	Candidatus-division-Zixibacteria-3-3	candidate division Zixibacteria	candidate division Zixibacteria bacterium RBG_16_43_9	ASM177535v1
Bacteria	Candidatus-Adlerbacteria-3-1	Candidatus Adlerbacteria	Candidatus Adlerbacteria bacterium GW2011_GWC1_50_9	ASM100420v1
Bacteria	Candidatus-Adlerbacteria-3-2	Candidatus Adlerbacteria	Candidatus Adlerbacteria bacterium GW2011_GWA1_54_10	ASM100466v1
Bacteria	Candidatus-Adlerbacteria-3-3	Candidatus Adlerbacteria	Candidatus Adlerbacteria bacterium GW2011_GWA2_54_12	ASM100472v1
Bacteria	Candidatus-Desantisbacteria-2-1	Candidatus Desantisbacteria	Candidatus Desantisbacteria bacterium CG1_02_38_46	ASM187101v1
Bacteria	Candidatus-Desantisbacteria-2-2	Candidatus Desantisbacteria	Candidatus Desantisbacteria bacterium CG1_02_49_89	ASM187107v1
Bacteria	Candidatus-Firestonebacteria-3-1	Candidatus Firestonebacteria	Candidatus Firestonebacteria bacterium RIFOXYA2_FULL_40_8	ASM177835v1
Bacteria	Candidatus-Firestonebacteria-3-2	Candidatus Firestonebacteria	Candidatus Firestonebacteria bacterium RIFOXYD2_FULL_39_29	ASM177837v1
Bacteria	Candidatus-Firestonebacteria-3-3	Candidatus Firestonebacteria	Candidatus Firestonebacteria bacterium RifOxyC12_full_39_7	ASM178034v1
Bacteria	Candidatus-Gracilibacteria-1-1	Candidatus Gracilibacteria	Gracilibacteria bacterium JGI 0000069-P22	ASM40472v1
Bacteria	Candidatus-Margulisbacteria-2-1	Candidatus Margulisbacteria	Candidatus Margulisbacteria bacterium GWF2_35_9	ASM178327v1
Bacteria	Candidatus-Margulisbacteria-2-2	Candidatus Margulisbacteria	Candidatus Margulisbacteria bacterium GWF2_38_17	ASM178451v1
Bacteria	Candidatus-Rokubacteria-3-1	Candidatus Rokubacteria	Candidatus Rokubacteria bacterium CSP1-6	ASM144338v1
Bacteria	Candidatus-Rokubacteria-3-2	Candidatus Rokubacteria	Candidatus Rokubacteria bacterium GWA2_70_23	ASM178839v1
Bacteria	Candidatus-Rokubacteria-3-3	Candidatus Rokubacteria	Candidatus Rokubacteria bacterium RIFCSPHIGHO2_12_FULL_73_22	ASM178847v1
Bacteria	Candidatus-Tectomicrobia-3-1	Candidatus Tectomicrobia	Candidatus *Entotheonella* sp.	v3
Bacteria	Candidatus-Tectomicrobia-3-2	Candidatus Tectomicrobia	Candidatus Tectomicrobia bacterium RIFCSPLOWO2_02_FULL_70_19	ASM179093v1
Bacteria	Candidatus-Tectomicrobia-3-3	Candidatus Tectomicrobia	Candidatus Tectomicrobia bacterium RIFCSPLOWO2_12_FULL_69_37	ASM179095v1

Domain	Code name of species	Phylum	Species	Accession number
Bacteria	Nitrospinae-3-1	Nitrospinae	*Nitrospina gracilis*	ASM34154v2
Bacteria	Nitrospinae-3-2	Nitrospinae	*Nitrospina* sp.	ASM104449v1
Bacteria	Nitrospinae-3-3	Nitrospinae	*Nitrospina* sp.	ASM104450v1
Bacteria	Unknown-30-1		bacteria symbiont BFo1 of *Frankliniella occidentalis*	ASM160254v1
Bacteria	Unknown-30-2		candidate division TM6 bacterium GW2011_GWF2_32_72	ASM98952v1
Bacteria	Unknown-30-3		candidate division TM6 bacterium GW2011_GWF2_37_49	ASM98995v1
Bacteria	Unknown-30-4		candidate division Kazan bacterium GW2011_GWC1_52_13	ASM100437v1
Bacteria	Unknown-30-5		candidate division Kazan bacterium GW2011_GWB1_52_7	ASM100444v1
Bacteria	Unknown-30-6		candidate division Kazan bacterium GW2011_GWA1_50_15	ASM102979v1
Bacteria	Unknown-30-7		Microgenomates group bacterium GW2011_GWD1_47_13	ASM100046v1
Bacteria	Unknown-30-8		Microgenomates group bacterium GW2011_GWF2_45_18	ASM100157v1
Bacteria	Unknown-30-9		Microgenomates group bacterium GW2011_GWF1_46_12	ASM100220v1
Bacteria	Unknown-30-10		*Vermiphilus pyriformis*	ASM38563v1
Bacteria	Unknown-30-11		bacterium JKG1	ASM52641v1
Bacteria	Unknown-30-12		Candidatus *Moduliflexus flocculans*	ASM73951v1
Bacteria	Unknown-30-13		Candidatus *Vecturithrix granuli*	ASM73953v1
Bacteria	Unknown-30-14		candidate division Kazan bacterium RBG_13_50_9	ASM177225v1
Bacteria	Unknown-30-15		bacterium LF-3	Guyana massiliensis
Bacteria	Unknown-30-16		bacterium MS4	Amazonia massiliensis
Bacteria	Unknown-30-17		candidate division WOR-1 bacterium RIFOXYA12_FULL_43_27	ASM177141v1
Bacteria	Unknown-30-18		candidate division WOR-1 bacterium RIFOXYA12_FULL_52_29	ASM177142v1
Bacteria	Unknown-30-19		candidate division WOR-1 bacterium RIFOXYA2_FULL_51_19	ASM177146v1
Bacteria	Candidatus Kapabacteria-1-1	Candidatus Kapabacteria	Candidatus *Kapabacteria* sp.	ASM189917v1
Bacteria	Chloroflexi-7	Chloroflexi	*Thermobaculum terrenum*	ASM2500v1
Bacteria	Unknown-30-22		PVC group bacterium (ex Bugula neritina AB1)	AB1_pvc

Domain	Code name of species	Phylum	Species	Accession number
Bacteria	Unknown-30-23		methanotrophic endosymbiont of *Bathymodiolus azoricus* (Menez Gwen)	BAZMOX
Bacteria	Unknown-30-24		Candidatus Melainabacteria bacterium MEL.A1	ASM176541v1
Bacteria	Unknown-30-25		Candidatus Melainabacteria bacterium GWA2_34_9	ASM178453v1
Bacteria	Unknown-30-26		Candidatus Melainabacteria bacterium GWF2_37_15	ASM178456v1
Bacteria	Unknown-30-27		Parcubacteria group bacterium RIFCSPHIGHO2_02_FULL_48_10b	ASM182439v1
Bacteria	Unknown-30-28		Parcubacteria group bacterium CG1_02_41_26	ASM187242v1
Bacteria	Unknown-30-29		candidate division Kazan bacterium RIFCSPHIGHO2_01_FULL_44_14	ASM177118v1
Bacteria	Unknown-30-30		candidate division TA06 bacterium DG_78	ASM130322v1
