# Supplementary material for: Reconstruction of the rRNA Sequences of LUCA, with Bioinformatic Implication of the Local Similarities Shared by Them
Source: Biology (Basel). 2022 May 29;11(6):837. doi: 10.3390/biology11060837 (PMC9219793; doi:10.3390/biology11060837)
Supplement: Supplementary file 1 [file biology-11-00837-s001.zip › Table S5.pdf]

Supplementary Table S5. Short fragments with the length of 11 in RY level.

Short fragments	Short fragments	N-Sf	N-Ec	Species							N-sp
RRRRRYRRYYY	GGGGAUAACCC	1-130	145		*P. abyssi*						1
RRRRRYRRYYY	GGAAACAACCC	2-1116	977	*E. coli*	*P. abyssi*						2
RRYRRRRYRRY	GGUGGGGUAAC	1-245	254	*E. coli*	*P. abyssi*	*S. cerevisiae*		*A. thaliana*			4
RRYRRRRYRRY	AACAAGGUAGC	1-1485	1499	*E. coli*	*P. abyssi*						2
RRYRRYYYRYY	AACGGCCCACC	1-253	262	*E. coli*	*P. abyssi*	*S. cerevisiae*		*A. thaliana*			4
RRYRRYYYRYY	AACAGCUCACU	2-1240	1095	*E. coli*	*P. abyssi*	*S. cerevisiae*	*P. solitarium*	*A. thaliana*	*D. melanogaster*	*H. sapiens*	**7**
RYYRYRRYRRY	GCCGCGGUAAU	1-491	524	*E. coli*	*P. abyssi*	*S. cerevisiae*	*P. solitarium*	*A. thaliana*	*D. melanogaster*	*H. sapiens*	**7**
RYYRYRRYRRY	GCUACAAUGGC	1-1218	1233	*E. coli*							1
RRRRYRYYRYY	GGGAUACUGCC	1-605	637								0
RRRRYRYYRYY	GAAACACCACC	2-2359	2168	*E. coli*	*P. abyssi*	*S. cerevisiae*	*P. solitarium*	*A. thaliana*	*D. melanogaster*	*H. sapiens*	**7**
YRRRRRYRRRR	UAGGGGUGAAA	1-654	686		*P. abyssi*	*S. cerevisiae*	*P. solitarium*	*A. thaliana*		*H. sapiens*	5
YRRRRRYRRRR	UAGGGGUGAAA	2-909	773	*E. coli*	*P. abyssi*	*S. cerevisiae*		*A. thaliana*	*D. melanogaster*	*H. sapiens*	**6**
YRRRRRYRRRR	CGGAAGUGAGA	2-1383	1243								0
YYRRRRRRYRY	CUGGGGAGUAC	1-855	883	*E. coli*	*P. abyssi*						2
YYRRRRRRYRY	UUAAAGAGUGC	2-1227	1082		*P. abyssi*	*S. cerevisiae*	*P. solitarium*	*A. thaliana*	*D. melanogaster*	*H. sapiens*	**6**
RYRRRRYYRRR	GCAAGGCUGAA	1-870	898	*E. coli*	*P. abyssi*	*S. cerevisiae*		*A. thaliana*	*D. melanogaster*	*H. sapiens*	**6**
RYRRRRYYRRR	GUGAAGCCGGG	2-837	702			*S. cerevisiae*	*P. solitarium*	*A. thaliana*		*H. sapiens*	4
YYYRRRRRRRY	CUCAAAGGAAU	1-882	910		*P. abyssi*	*S. cerevisiae*	*P. solitarium*	*A. thaliana*	*D. melanogaster*	*H. sapiens*	**6**
YYYRRRRRRRY	UCUAGGGGGAC	1-1132	1148		*P. abyssi*	*S. cerevisiae*	*P. solitarium*	*A. thaliana*		*H. sapiens*	5
RYYRRYRRRRR	AUUGACGGGGG	1-891	919	*E. coli*	*P. abyssi*	*S. cerevisiae*	*P. solitarium*	*A. thaliana*	*D. melanogaster*	*H. sapiens*	**7**
RYYRRYRRRRR	GCCGAUGAAGG	2-45	45		*P. abyssi*						1
RYRRYYYRRYY	GCGGUUUAAUU	1-924	951	*E. coli*	*P. abyssi*	*S. cerevisiae*	*P. solitarium*	*A. thaliana*	*D. melanogaster*	*H. sapiens*	**7**
RYRRYYYRRYY	GCAGUUUGACU	2-2431	2239	*E. coli*	*P. abyssi*						2
RYRRYYYRRYY	ACAGUUCGGUC	2-2796	2600	*E. coli*							1
RRRRRYYYYRY	AGGAACCUUAC	1-947	974	*E. coli*	*P. abyssi*					*H. sapiens*	3
RRRRRYYYYRY	AAAGACCCCGU	2-2249	2058	*E. coli*	*P. abyssi*	*S. cerevisiae*	*P. solitarium*	*A. thaliana*		*H. sapiens*	**6**
YYRRYYYRYRY	UCAGCUCGUGC	1-1041	1065	*E. coli*	*P. abyssi*						2
YYRRYYYRYRY	CCGACCUGCAC	2-2148	1957	*E. coli*	*P. abyssi*						2
RYYRYRRRRYR	GCCGUGAGGUG	1-1050	1074	*E. coli*	*P. abyssi*						2
RYYRYRRRRYR	GCCGUAGAGUG	2-2508	2315		*P. abyssi*						1
RYYRYRRRRYR	GUCGUGAGACA	2-2788	2592	*E. coli*	*P. abyssi*	*S. cerevisiae*	*P. solitarium*	*A. thaliana*	*D. melanogaster*	*H. sapiens*	**7**
RRRRYYRYRYY	GGAGCUAAUCC	1-1258	1273								0
RRRRYYRRYYY	GAGGUUGGCUU	2-1201	1056								0
RYYRYRRYYYR	GCUGCAACUCG	1-1298	1313		*P. abyssi*						1
RYYRYRRYYYR	ACUGCAGCCUG	2-2268	2077	*E. coli*	*P. abyssi*						2
RRRYYRYRRYR	AAGUCGUAACA	1-1478	1492	*E. coli*	*P. abyssi*	*S. cerevisiae*		*A. thaliana*	*D. melanogaster*	*H. sapiens*	**6**
RRRYYRYRRYR	AAGCUGCGAUA	2-63	63		*P. abyssi*						1
RRRYYRYRRYR	AGGUCGCAGUG	2-1937	1748		*P. abyssi*						1
RRYYRYYYYYY	GAUCACCUCCU	1-1516	1530	*E. coli*							1
RRYYRYYYYYY	AGCCAUCCUUU	2-1218	1073		*P. abyssi*						1
RRYYYYYRRRY	GAUUUCCGAAU	2-109	110	*E. coli*	*P. abyssi*						2
RRYYYYYRRRY	GGUCCCUAAGU	2-1141	1002								0
YRYYYYRRYRY	CAUCUUAGUAC	2-200	198		*P. abyssi*						1
YRYYYYRRYRY	CACCUCGAUGU	2-2692	2496	*E. coli*							1

Short fragments	Short fragments	N-Sf	N-Ec	Species							N-sp
RRRRRRRRRRR	AGGAAAAGAAA	2-215	213	*E. coli*	*P. abyssi*	*S. cerevisiae*	*P. solitarium*	*A. thaliana*	*D. melanogaster*	*H. sapiens*	**7**
RRRRRRRRRRR	GGGAGAAGGGG	2-1884	1695		*P. abyssi*						1
YRRYRRYRRRY	UAGCGGCGAGC	2-245	243	*E. coli*		*S. cerevisiae*	*P. solitarium*	*A. thaliana*	*D. melanogaster*	*H. sapiens*	**6**
YRRYRRYRRRY	CGAUAGCGAAC	2-495	445	*E. coli*	*P. abyssi*	*S. cerevisiae*	*P. solitarium*	*A. thaliana*	*D. melanogaster*		**6**
RRRRRRYRRYR	AGAGGGUGAUA	2-370	322		*P. abyssi*						1
RRRRRRYRRYR	GGGGGACAGUG	2-2412	2220		*P. abyssi*						1
RYRRRRRYYYR	GUGGGAAUCUG	2-446	396								0
RYRRRRRYYYR	GUGGAGGCCCG	2-858	723		*P. abyssi*	*S. cerevisiae*	*P. solitarium*	*A. thaliana*		*H. sapiens*	5
RRRYYRRYRYY	GAACUAGUACC	2-502	452	*E. coli*	*P. abyssi*						2
RRRYYRRYRYY	AGGUUAAUAUU	2-1531	1387	*E. coli*	*P. abyssi*		*P. solitarium*				3
RYYYRRRRYYR	GCCUGAAACCA	2-558	508	*E. coli*	*P. abyssi*		*P. solitarium*	*A. thaliana*			4
RYYYRRRRYYR	ACCCGAAACCG	2-805	670		*P. abyssi*						1
YYYYRRRRYRR	CCCCGAAAUAG	2-950	814								0
YYYYRRRRYRR	UCCUAAGGUAG	2-2114	1923	*E. coli*	*P. abyssi*	*S. cerevisiae*	*P. solitarium*	*A. thaliana*	*D. melanogaster*	*H. sapiens*	**7**
YRRYRYRRRYR	CGACACAGGUG	2-1797	1612	*E. coli*	*P. abyssi*						2
YRRYRYRRRYR	UAGCGUAGGUG	2-2304	2113		*P. abyssi*						1
RRRRYYYRRYR	GGAACUCGGCA	2-1853	1666		*P. abyssi*						1
RRRRYYYRRYR	GGGACCCGGUG	2-2193	2002	*E. coli*	*P. abyssi*						2
YRRRRRYRYRR	CAAAAACACAG	2-1972	1782	*E. coli*	*P. abyssi*						2
YRRRRRYRYRR	CGGAGGUGUAA	2-3034	2841		*P. abyssi*						1
RYRRYYRYRRY	GUAACUAUAAC	2-2101	1910	*E. coli*	*P. abyssi*	*S. cerevisiae*	*P. solitarium*	*A. thaliana*	*D. melanogaster*	*H. sapiens*	**7**
RYRRYYRYRRY	GUGGCCAUAGC	5-7	4473	*E. coli*	*P. abyssi*						2
YRRRRRYRYYY	CGGAGGCGCCC	2-2468	2275		*P. abyssi*				*D. melanogaster*		2
YRRRRRYRYYY	UGAAAGCAUCU	2-2943	2746	*E. coli*	*P. abyssi*						2
RRRYYRRRYYY	AAGCCGGGCUU	2-2573	2377								0
RRRYYRRRYYY	GAGCUGGGUUC	2-2772	2576	*E. coli*	*P. abyssi*	*S. cerevisiae*	*P. solitarium*	*A. thaliana*	*D. melanogaster*	*H. sapiens*	**7**
RRRYYRRRYYY	AAGUUAAGCCC	5-52	4518		*P. abyssi*						1
YYYRYYYRYYR	UUCGCCCAUUA	2-2750	2554		*P. abyssi*	*S. cerevisiae*	*P. solitarium*	*A. thaliana*		*H. sapiens*	5
YYYRYYYRYYR	UCUAUCUGCUG	2-2807	2611	*E. coli*							1
RYYYYYRRYRY	GCCCCUAGUAC	2-2845	2648	*E. coli*	*P. abyssi*						2
RYYYYYRRYRY	ACCUCUGGUGU	2-2876	2679								0

Short fragments were searched from the 16S, 23S, and 5S rRNA sequences of LUCA, which were converted from 'A', 'G' to 'R' and converted from 'U', 'C' to 'Y'. N-Sf: The code number of the first nucleotide of the short fragment. The number before '-' is the location where 1' is for 16S rRNA and '2' is for 23S rRNA. The number after '-' refers to the number of ancestral sequences with gaps. N-Ec: The number of the orthologous nucleotide of the short fragment's first nucleotide in the rRNAs of E. coli. Species: The purine – pyrimidine arrangements of short fragments are the same as the orthologous sequences of the listed species. N-sp: The number of listed species. The short fragments with red, magenta, and black fonts correspond to those marked by red, magenta, and gray, respectively, in Figures 4 and 5.
