# Supplementary material for: Reconstruction of the rRNA Sequences of LUCA, with Bioinformatic Implication of the Local Similarities Shared by Them
Source: Biology (Basel). 2022 May 29;11(6):837. doi: 10.3390/biology11060837 (PMC9219793; doi:10.3390/biology11060837)
Supplement: Supplementary file 1 [file biology-11-00837-s001.zip › Table S6.pdf]

Supplementary Table S6. Functions of nucleotides in short fragments (RY level and 11 in length).

Function	N-Box	N-F18	N-F57	N-NF75	References
interaction with tRNA in A-, P-, and E-sites	1, 2, 3, 4, 5, 6, 8, 9, 10	G529(1-491), G530(1-491), U531(1-491), G2583(2-2772), A2602(2-2788), A1492(1-1478), A1493(1-1478), G1494(1-1478), A532(1-491), A1916(2-2101), A1918(2-2101), U1926(2-2114), U2585(2-2772), G926(1-891), U956(1-924), U2584(2-2772), A2169(2-2359), A1913(2-2101)	U2609(2-2796), U2506(2-2692), U2555(2-2750), C2501(2-2692), G2603(2-2796), G2505(2-2692), G693(1-654), G2116(2-2304)	G2553(23S), C2507(23S), C2573(23S), A2451(23S), C2452(23S), C2493(23S), G2494(23S), G1041(23S), G1068(23S), G1071(23S), C1941(23S), C2254(23S), A2439(23S), C936(16S), C1378(16S), A1408(16S), G2251(23S), A2450(23S), G2252(23S), G2253(23S), A2439(23S), A794(16S), C795(16S), G966(16S), G1338(16S), A1339(16S), U1381(16S), C1399(16S), C1400(16S), G1401(16S), G140(16S), G2112(23S), C2394(23S), U1376(16S), C1378(16S), C1909(23S), U1943(23S), C1942(23S)	[25- 28, 30-33]
interaction with mRNA	1	A532(1-491)	G693(1-654), G1156(1-1132), C1533(1-1516), U1532(1-1516), 1530(1-1516), 1534(1-1516), 1535(1-1516), 1536(1-1516), 1537(1-1516), 1538(1-1516), 1539(1-1516), 1540(1-1516)	U887(23S), A412(16S), G413(16S), U420(16S), G436(16S), G497(16S), 665(16S), U723(16S), A794(16S), C795(16S), G818(16S), A845(16S), 1052(16S), A1167(16S), A1196(16S), G1300(16S), U1381(16S), C1395(16S), G1338(16S), 1360(16S), 1402(16S), 1541(16S), 1542(16S)	[27, 34-37]
interaction with mRNA–tRNA minihelix	1,4	A1492(1-1478), A1493(1-1478), G530(1-491)			[38]
interaction with nascent peptide	7,9	A2062(2-2249), U2585(2-2772)	U2506(2-2692), U2609(2-2796), A1614(2-1797)	U1781(23S), A750(23S)	[39-40]
monitoring the codon–anticodon pairing and	4	A1492(1-1478), A1493(1-1478)		G517(16S)	[41-46]

Function	N-Box	N-F18	N-F57	N-NF75	References
maintaining translational fidelity					
co-translational monitoring of nascent peptide chains inside the exit tunnel	7	A2062(2-2249)			[43]
Recognition of stop codon	1,4, 5	A1913(2-2101), G530(1-491), A1493(1-1478)			[47]
nascent peptide tunnel	7	A2058(2-2249), A2059(2-2249), A2060(2-2249), G2061(2-2249), A2062(2-2249), C2063(2-2249)			[30, 48]
taking part in the peptide release	9,10	U2585(2-2772), A2602(2-2788)			[30, 49]
Forming base pairs with other functional nucleotides	1, 7	G2061(2-2249), C2063(2-2249), G530(1-491)	C2499(2-2692)	A2450(23S), U2457(23S), G2447(23S), A2453(23S)	[25, 30, 52]
interacting with GTPase factors (EF-G, EF-Tu, IF2, RF3)			2653(2-2845), 2654(2-2845), 2655(2-2845), 2656(2-2845), 2657(2-2845), 2658(2-2845)	2659(23S), 2660(23S), 2661(23S), 2662(23S), 2663(23S), 2664(23S), 2665(23S), 2666(23S), 2667(23S)	[53-58]
Inter-subunit bridges	4, 5, 9,10,11	900(1-870), 901(1-870), 1912(2-2101), 1913(2-2101), 1923(2-2114), 1928(2-2114), 1929(2-2114), 1932(2-2114), 1933(2-2114), A1493(1-1478), A2602(2-2788), U2585(2-2772)	1495(1-1478), 1496(1-1478), 1702(2-1884), 1703(2-1884), 1704(2-1884), 1705(2-1884), 1960(2-2148), 1961(2-2148), 1962(2-2148), U2506(2-2692)	339(16S), 340(16S), 345(16S), 346(16S), 669(16S), 670(16S), 671(16S), 698(16S), 699(16S), 701(16S), 702(16S), 703(16S), 712(16S), 713(16S), 714(16S), 762(16S), 770(16S), 771(16S), 772(16S), 773(16S), 774(16S), 775(16S), 776(16S), 783(16S), 784(16S), 785(16S), 786(16S), 791(16S), 792(16S), 1409(16S), 1410(16S), 1418(16S), 1419(16S), 1420(16S), 1421(16S), 1422(16S), 1423(16S), 1429(16S), 1430(16S), 1431(16S),	[50-51]

Function	N-Box	N-F18	N-F57	N-NF75	References
				1432(16S), 1433(16S), 1463(16S), 1464(16S), 1465(16S), 1473(16S), 1474(16S), 1475(16S), 1476(16S), 1483(16S), 1484(16S), 1485(16S), 1486(16S), 1514(16S), 1515(16S), 1516(16S), 714(23S), 715(23S), 716(23S), 881(23S), 882(23S), 883(23S), 884(23S), 885(23S), 891(23S), 892(23S), 1689(23S), 1690(23S), 1718(23S), 1719(23S), 1793(23S), 1794(23S), 1830(23S), 1831(23S), 1832(23S), 1833(23S), 1834(23S), 1836(23S), 1837(23S), 1838(23S), 1847(23S), 1848(23S), 1896(23S), 1897(23S), 1922(23S), 1948(23S), 1949(23S), 1950(23S), 1951(23S), 1988(23S), 1989(23S), A2572(23S)	

N-Box: Boxed number corresponding to that in Figure 5. N-F18: Functional nucleotide sites contained in the 18 short fragments. N-F57: Functional nucleotide sites contained in the other 57 short fragments. N-F18 and N-F57: The part outside the bracket is the number in the 16S and 23S rRNAs of E. coli. The part inside the bracket is the code number of the corresponding short fragment's first nucleotide. The number before '-' is the location where '1' is for 16S rRNA and '2'is for 23S rRNA. The number after '-' refers to the number of ancestral sequences with gaps. N-NF75: Positions of functional nucleotide sites not contained in the 75 short fragments.
