# Supplementary material for: Reconstruction of the rRNA Sequences of LUCA, with Bioinformatic Implication of the Local Similarities Shared by Them
Source: Biology (Basel). 2022 May 29;11(6):837. doi: 10.3390/biology11060837 (PMC9219793; doi:10.3390/biology11060837)
Supplement: Supplementary file 1 [file biology-11-00837-s001.zip › Table S8.pdf]

Supplementary Table S8. Short fragments with the length of 6 in AUGC level.

Short fragments	N-Sf	N-Ec	Species							N-sp
AAGUCG	1-60	59	*E. coli*							1
AAGUCG	1-1478	1492	*E. coli*	*P. abyssi*	*S. cerevisiae*		*A. thaliana*	*D. melanogaster*	*H. sapiens*	6
GCGAAC	1-89	105								0
GCGAAC	1-746	778		*P. abyssi*						1
GUAACA	1-101	117		*P. abyssi*						1
GUAACA	1-1483	1497	*E. coli*	*P. abyssi*	*S. cerevisiae*		*A. thaliana*	*D. melanogaster*	*H. sapiens*	6
GUAACA	2-1238	1093		*P. abyssi*	*S. cerevisiae*	*P. solitarium*	*A. thaliana*	*D. melanogaster*	*H. sapiens*	6
GGGAUA	1-131	146	*E. coli*	*P. abyssi*						2
GGGAUA	1-605	637		*P. abyssi*						1
GGGAUA	2-2641	2445	*E. coli*	*P. abyssi*	*S. cerevisiae*	*P. solitarium*	*A. thaliana*	*D. melanogaster*	*H. sapiens*	7
UAACCC	1-135	150		*P. abyssi*						1
UAACCC	2-2388	2197		*P. abyssi*						1
GGUGGG	1-245	254	*E. coli*	*P. abyssi*						2
GGUGGG	2-2311	2120	*E. coli*							1
GGUGGG	2-2426	2234	*E. coli*	*P. abyssi*	*S. cerevisiae*	*P. solitarium*	*A. thaliana*		*H. sapiens*	6
UGCCAG	1-483	516	*E. coli*		*S. cerevisiae*	*P. solitarium*	*A. thaliana*	*D. melanogaster*	*H. sapiens*	6
UGCCAG	1-1102	1126	*E. coli*	*P. abyssi*						2
CGGUAA	1-495	528	*E. coli*	*P. abyssi*	*S. cerevisiae*	*P. solitarium*	*A. thaliana*	*D. melanogaster*	*H. sapiens*	7
CGGUAA	2-2086	1895	*E. coli*	*P. abyssi*						2
GGUUUG	1-554	587	*E. coli*							1
GGUUUG	2-2685	2489								0
GCGGCU	1-581	614								0
GCGGCU	1-1509	1523								0
UAGGGG	1-654	686		*P. abyssi*						1
UAGGGG	2-909	773		*P. abyssi*	*S. cerevisiae*		*A. thaliana*	*D. melanogaster*	*H. sapiens*	5
UAGGGG	2-1328	1188	*E. coli*							1
GGUGAA	1-658	690	*E. coli*	*P. abyssi*	*S. cerevisiae*	*P. solitarium*	*A. thaliana*	*D. melanogaster*	*H. sapiens*	7
GGUGAA	2-836	701		*P. abyssi*	*S. cerevisiae*					2
GGUGAA	2-913	777	*E. coli*	*P. abyssi*						2
GGUGAA	2-2200	2009								0
GGUGAA	2-2217	2026								0
CGGGAG	1-676	708								0
CGGGAG	2-1076	937	*E. coli*							1
CGGGAG	2-1883	1694	*E. coli*	*P. abyssi*						2
CAGUGC	1-823	851								0
CAGUGC	2-2028	1838		*P. abyssi*	*S. cerevisiae*	*P. solitarium*	*A. thaliana*	*D. melanogaster*	*H. sapiens*	6
CGAAGC	1-829	857								0
CGAAGC	2-2078	1887	*E. coli*							1
CGAAGC	2-2959	2762		*P. abyssi*						1
CUGGGG	1-855	883	*E. coli*	*P. abyssi*						2
CUGGGG	2-2440	2248	*E. coli*	*P. abyssi*	*S. cerevisiae*	*P. solitarium*	*A. thaliana*	*D. melanogaster*	*H. sapiens*	7
AGUACG	1-861	889	*E. coli*	*P. abyssi*						2
AGUACG	2-2851	2654	*E. coli*	*P. abyssi*	*S. cerevisiae*	*P. solitarium*	*A. thaliana*	*D. melanogaster*	*H. sapiens*	7
GUGGAG	1-915	942	*E. coli*	*P. abyssi*	*S. cerevisiae*	*P. solitarium*	*A. thaliana*	*D. melanogaster*	*H. sapiens*	7
GUGGAG	2-858	723			*S. cerevisiae*	*P. solitarium*			*H. sapiens*	3

GUGGAG	2-2258	2067								0
GUGGAG	2-2345	2154		*P. abyssi*						1
CAGCUC	1-1042	1066	*E. coli*	*P. abyssi*						2
CAGCUC	2-1242	1097		*P. abyssi*						1
CGUGAG	1-1052	1076		*P. abyssi*						1
CGUGAG	2-512	462	*E. coli*	*P. abyssi*		*P. solitarium*		*D. melanogaster*		4
CGUGAG	2-2790	2594	*E. coli*	*P. abyssi*	*S. cerevisiae*	*P. solitarium*	*A. thaliana*	*D. melanogaster*	*H. sapiens*	7
UAACGA	1-1076	1100		*P. abyssi*	*S. cerevisiae*	*P. solitarium*	*A. thaliana*	*D. melanogaster*	*H. sapiens*	6
UAACGA	2-2167	1976		*P. abyssi*	*S. cerevisiae*		*A. thaliana*	*D. melanogaster*		4
CGGAGG	1-1158	1173		*P. abyssi*						1
CGGAGG	2-212	210			*S. cerevisiae*	*P. solitarium*	*A. thaliana*		*H. sapiens*	4
AUGGCC	1-1189	1204	*E. coli*							1
AUGGCC	1-1224	1239								0
GCUACA	1-1207	1222	*E. coli*	*P. abyssi*						2
GCUACA	1-1218	1233	*E. coli*	*P. abyssi*	*S. cerevisiae*	*P. solitarium*	*A. thaliana*	*D. melanogaster*	*H. sapiens*	7
CAGUUC	1-1283	1298		*P. abyssi*						1
CAGUUC	2-2797	2601	*E. coli*							1
GCCGUA	1-1494	1508		*P. abyssi*						1
GCCGUA	2-730	622		*P. abyssi*						1
GCCGUA	2-2508	2315		*P. abyssi*						1
AGCCGA	2-44	44								0
AGCCGA	2-341	294		*P. abyssi*						1
UAAGCC	2-72	72		*P. abyssi*						1
UAAGCC	5-56	56		*P. abyssi*						1
CCGAAU	2-114	115	*E. coli*	*P. abyssi*						2
CCGAAU	2-1051	914								0
GGGGAA	2-188	188	*E. coli*	*P. abyssi*						2
GGGGAA	5-21	21								0
GAAACA	2-196	194	*E. coli*	*P. abyssi*						2
GAAACA	2-1117	978	*E. coli*							1
AGUACC	2-206	204	*E. coli*	*P. abyssi*						2
AGUACC	2-421	371		*P. abyssi*						1
AGUACC	2-507	457	*E. coli*	*P. abyssi*		*P. solitarium*	*A. thaliana*	*D. melanogaster*	*H. sapiens*	6
GAAAAG	2-217	215	*E. coli*	*P. abyssi*	*S. cerevisiae*	*P. solitarium*	*A. thaliana*	*D. melanogaster*	*H. sapiens*	7
GAAAAG	2-526	476	*E. coli*	*P. abyssi*	*S. cerevisiae*		*A. thaliana*	*D. melanogaster*	*H. sapiens*	6
GAAAAG	2-1751	1568	*E. coli*	*P. abyssi*						2
AAGCGA	2-230	228		*P. abyssi*						1
AAGCGA	2-740	632								0
AGUAGC	2-243	241	*E. coli*					*D. melanogaster*		2
AGUAGC	2-1405	1265		*P. abyssi*	*S. cerevisiae*	*P. solitarium*	*A. thaliana*	*D. melanogaster*	*H. sapiens*	6
GGCGAG	2-249	247	*E. coli*		*S. cerevisiae*	*P. solitarium*	*A. thaliana*	*D. melanogaster*	*H. sapiens*	6
GGCGAG	2-1496	1355	*E. coli*							1
GCGAAA	2-254	252						*D. melanogaster*		1
GCGAAA	2-390	342								0
GCGAAA	2-736	628								0
GCGAAA	2-2569	2373	*E. coli*							1
GGAAAG	2-355	307	*E. coli*				*A. thaliana*			2
GGAAAG	2-518	468	*E. coli*	*P. abyssi*	*S. cerevisiae*	*P. solitarium*		*D. melanogaster*	*H. sapiens*	6
AGAGGG	2-370	322		*P. abyssi*	*S. cerevisiae*	*P. solitarium*	*A. thaliana*			4

AGAGGG	2-1112	973	*E. coli*	*P. abyssi*						2
UGAUAG	2-376	328		*P. abyssi*						1
UGAUAG	2-3026		*E. coli*							1
CUGAAA	2-560	510	*E. coli*	*P. abyssi*						2
CUGAAA	2-2942	2745	*E. coli*							1
UUGAAG	2-672	568								0
UUGAAG	2-2322	2131	*E. coli*							1
AGACCC	2-803	668	*E. coli*	*P. abyssi*						2
AGACCC	2-2251	2060	*E. coli*	*P. abyssi*	*S. cerevisiae*	*P. solitarium*	*A. thaliana*	*D. melanogaster*	*H. sapiens*	7
CCGAAA	2-807	672	*E. coli*	*P. abyssi*	*S. cerevisiae*	*P. solitarium*	*A. thaliana*	*D. melanogaster*	*H. sapiens*	7
CCGAAA	2-952	816	*E. coli*							1
CCGAAA	2-1268	1123		*P. abyssi*			*A. thaliana*			2
GCCGGG	2-842	707								0
GCCGGG	2-2575	2379								0
AGCUGG	2-940	804	*E. coli*	*P. abyssi*	*S. cerevisiae*	*P. solitarium*	*A. thaliana*	*D. melanogaster*	*H. sapiens*	7
AGCUGG	2-2773	2577	*E. coli*	*P. abyssi*	*S. cerevisiae*	*P. solitarium*	*A. thaliana*	*D. melanogaster*	*H. sapiens*	7
GGGUAG	2-994	857	*E. coli*	*P. abyssi*						2
GGGUAG	2-2914	2717								0
UAAGGU	2-1138	999	*E. coli*		*S. cerevisiae*					2
UAAGGU	2-2117	1926	*E. coli*	*P. abyssi*	*S. cerevisiae*	*P. solitarium*	*A. thaliana*	*D. melanogaster*	*H. sapiens*	7
CAUCCU	2-1221	1076		*P. abyssi*						1
CAUCCU	2-2713	2517	*E. coli*	*P. abyssi*						2
UUAAAG	2-1227	1082	*E. coli*	*P. abyssi*						2
UUAAAG	2-2758	2562	*E. coli*	*P. abyssi*						2
GAGUGC	2-1232	1087		*P. abyssi*						1
GAGUGC	2-2514	2321		*P. abyssi*						1
CUAAGG	2-1492	1351	*E. coli*							1
CUAAGG	2-1824	1638		*P. abyssi*						1
ACAGGU	2-1529	1385	*E. coli*							1
ACAGGU	2-1801	1616	*E. coli*	*P. abyssi*						2
UCUAAG	2-1629	1466								0
UCUAAG	2-2951	2754	*E. coli*	*P. abyssi*	*S. cerevisiae*		*A. thaliana*	*D. melanogaster*	*H. sapiens*	6
GUAACU	2-1876	1687	*E. coli*	*P. abyssi*	*S. cerevisiae*		*A. thaliana*	*D. melanogaster*	*H. sapiens*	6
GUAACU	2-2101	1910	*E. coli*	*P. abyssi*	*S. cerevisiae*	*P. solitarium*	*A. thaliana*	*D. melanogaster*	*H. sapiens*	7
AAAGGU	2-2460	2267		*P. abyssi*						1
AAAGGU	2-2479	2286								0
CGCCCA	2-2474	2281								0
CGCCCA	2-2752	2556								0
GUUCAG	2-2779	2583								0
GUUCAG	2-3065	2864								0
GGUGUA	2-2882	2685							*H. sapiens*	1
GGUGUA	2-3038	2845								0

Short fragments were searched from the 16S, 23S, and 5S rRNA sequences of LUCA at the AUGC level. N-Sf: The code number of the first nucleotide of the short fragment. The number before '-' is the location where '1' is for 16S rRNA and '2' is for 23S rRNA. The number after '-' refers to the number of ancestral sequences with gaps. N-Ec: The number of orthologous nucleotides of the short fragment's first nucleotide in the rRNAs of E. coli. Species: The sequences of short fragments are the same as the orthologous sequences

of the listed species. N-sp: The number of listed species. The short fragments with red, magenta, and black fonts correspond to the ones marked by red, magenta, and gray, respectively, in Figure S6.
