# Supplementary material for: Reconstruction of the rRNA Sequences of LUCA, with Bioinformatic Implication of the Local Similarities Shared by Them
Source: Biology (Basel). 2022 May 29;11(6):837. doi: 10.3390/biology11060837 (PMC9219793; doi:10.3390/biology11060837)
Supplement: Supplementary file 1 [file biology-11-00837-s001.zip › Table S9.pdf]

**Supplementary Table S9. Functions of nucleotides in short fragments (AUGC level and 6 in length).**

| **Function** | **N-F136** |
|---|---|
| interaction with tRNA in A-, P-, and E-sites | 529(**1-495**), 530(**1-495**), 531(**1-495**), 532(**1-495**), 693(**1-658**), 1492(**1-1478**), 1493(**1-1478**), 1494(**1-1478**), 1913(**2-2101**), 1926(**2-2117**), 2251(**2-2440**), 2252(**2-2440**), 2253(**2-2440**), 2450(**2-2641**), 2493(2-2685), 2494(2-2685), 2583(2-2779), 2584(2-2779), 2585(2-2779), 2602(2-2797), 2603(2-2797) |
| interaction with mRNA | 530(**1-495**), 532(**1-495**), 693(**1-658**), 1300(1-1283) |
| interaction with mRNA–tRNA minihelix | 530(**1-495**), 1492(**1-1478**), 1493(**1-1478**) |
| interaction with nascent peptide | 2062(**2-2251**), 2585(2-2779) |
| monitoring the codon–anticodon pairing and maintaining translational fidelity | 517(**1-483**), 1492(**1-1478**), 1493(**1-1478**) |
| co-translational monitoring of nascent peptide chains inside the exit tunnel | 2062(**2-2251**) |
| Recognition of stop codon | 530(**1-495**), 1493(**1-1478**), 1913(**2-2101**) |
| nascent peptide tunnel | 2060(**2-2251**), 2061(**2-2251**), 2062(**2-2251**), 2063(**2-2251**) |
| taking part in the peptide release | 2585(2-2779), 2602(2-2797), |
| Forming base pairs with other functional nucleotides | 530(**1-495**), 2061(**2-2251**), 2063(**2-2251**), 2447(**2-2641**), 2450(**2-2641**) |
| interacting with GTPase factors (EF-G, EF-Tu, IF2, RF3) | 2654(**2-2851**), 2655(**2-2851**), 2656(**2-2851**), 2657(**2-2851**), 2658(**2-2851**), 2659(**2-2851**) |
| Inter-subunit bridges | 712(1-676), 713(1-676), 783(1-746), 1493(**1-1478**), 1495(**1-1478**), 1496(**1-1478**), 1689(**2-1876**), 1690(**2-1876**), 1838(**2-2028**), 1896(2-2086), 1897(2-2086), 1912(**2-2101**), 1913(**2-2101**), 1928(**2-2117**), 1929(**2-2117**), 2585(2-2779), 2602(2-2797) |

N-F136: Functional nucleotide sites contained in the 136 short fragments. The parts outside the bracket refer to the functional nucleotide site in the 16S and 23S rRNAs of E. coli. The part inside the bracket is the code number of the corresponding short fragment's first nucleotide. The bold code number marks the short fragments which were conserved across 5 or 6 kingdoms.
