# Supplementary figures and images for: Reconstruction of the rRNA Sequences of LUCA, with Bioinformatic Implication of the Local Similarities Shared by Them
Source: Biology (Basel). 2022 May 29;11(6):837. doi: 10.3390/biology11060837 (PMC9219793; doi:10.3390/biology11060837)

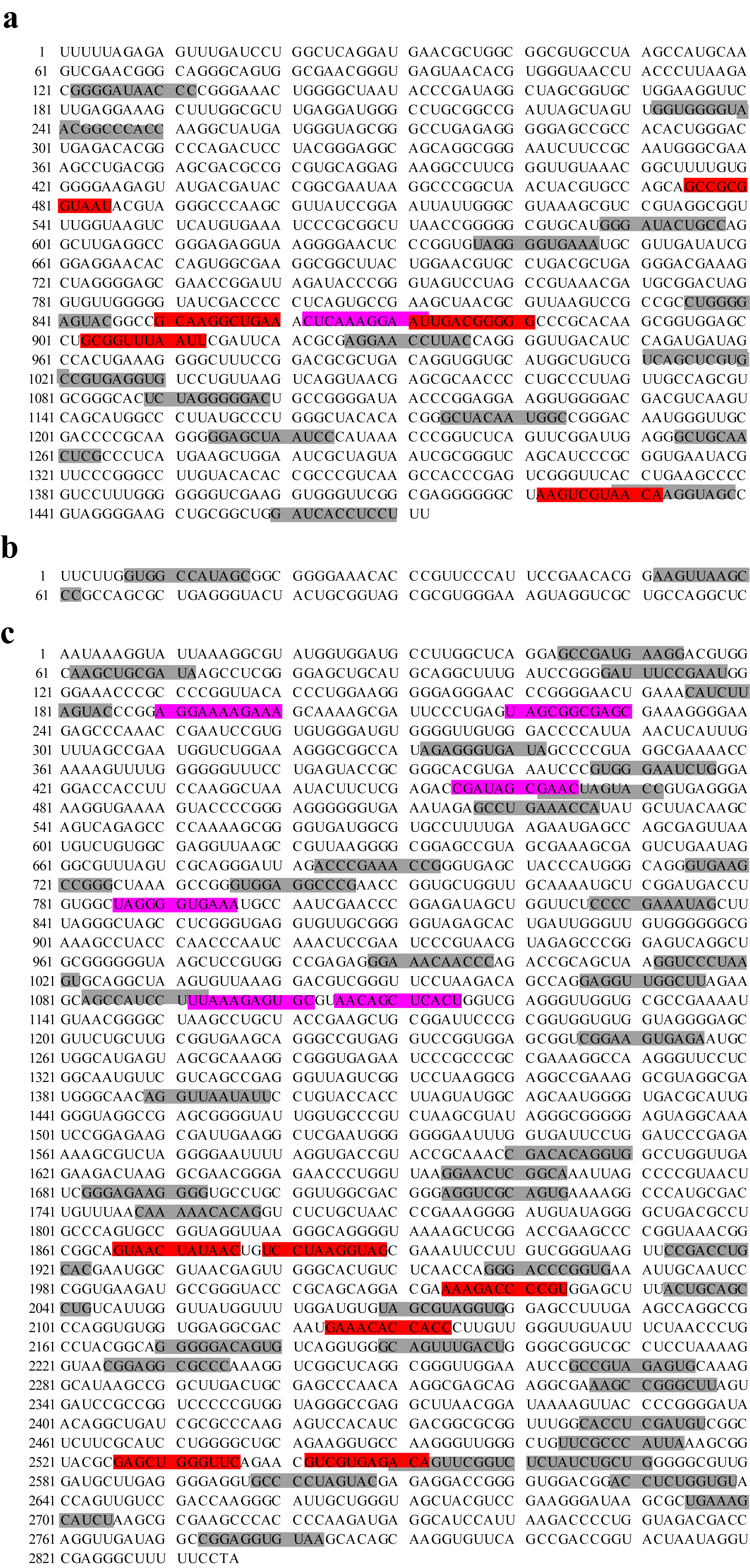

Supplement: Supplementary file 1 [file biology-11-00837-s001.zip › Figure S2.tif]

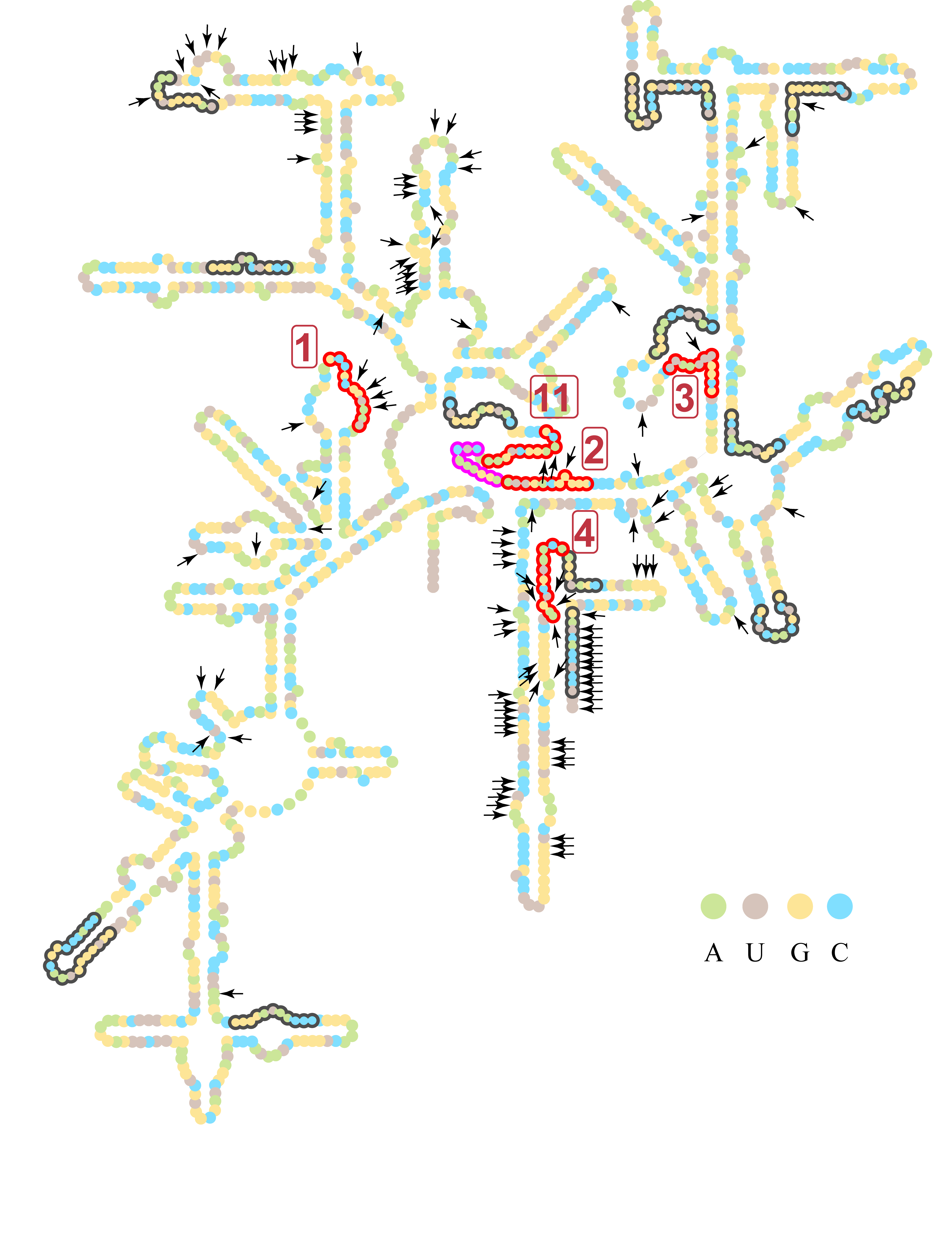

Supplement: Supplementary file 1 [file biology-11-00837-s001.zip › Figure S3.tif]

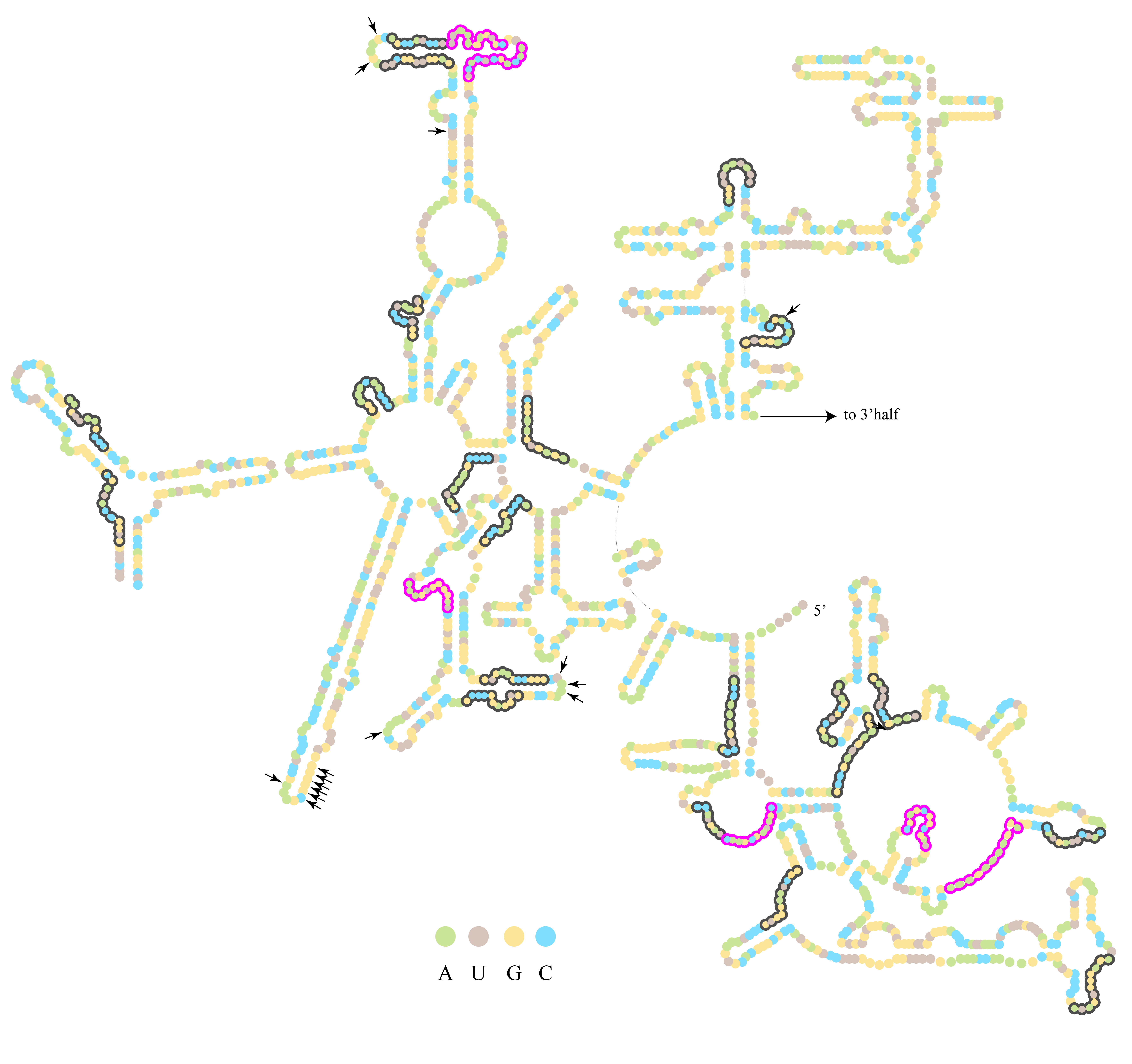

Supplement: Supplementary file 1 [file biology-11-00837-s001.zip › Figure S4.tif]

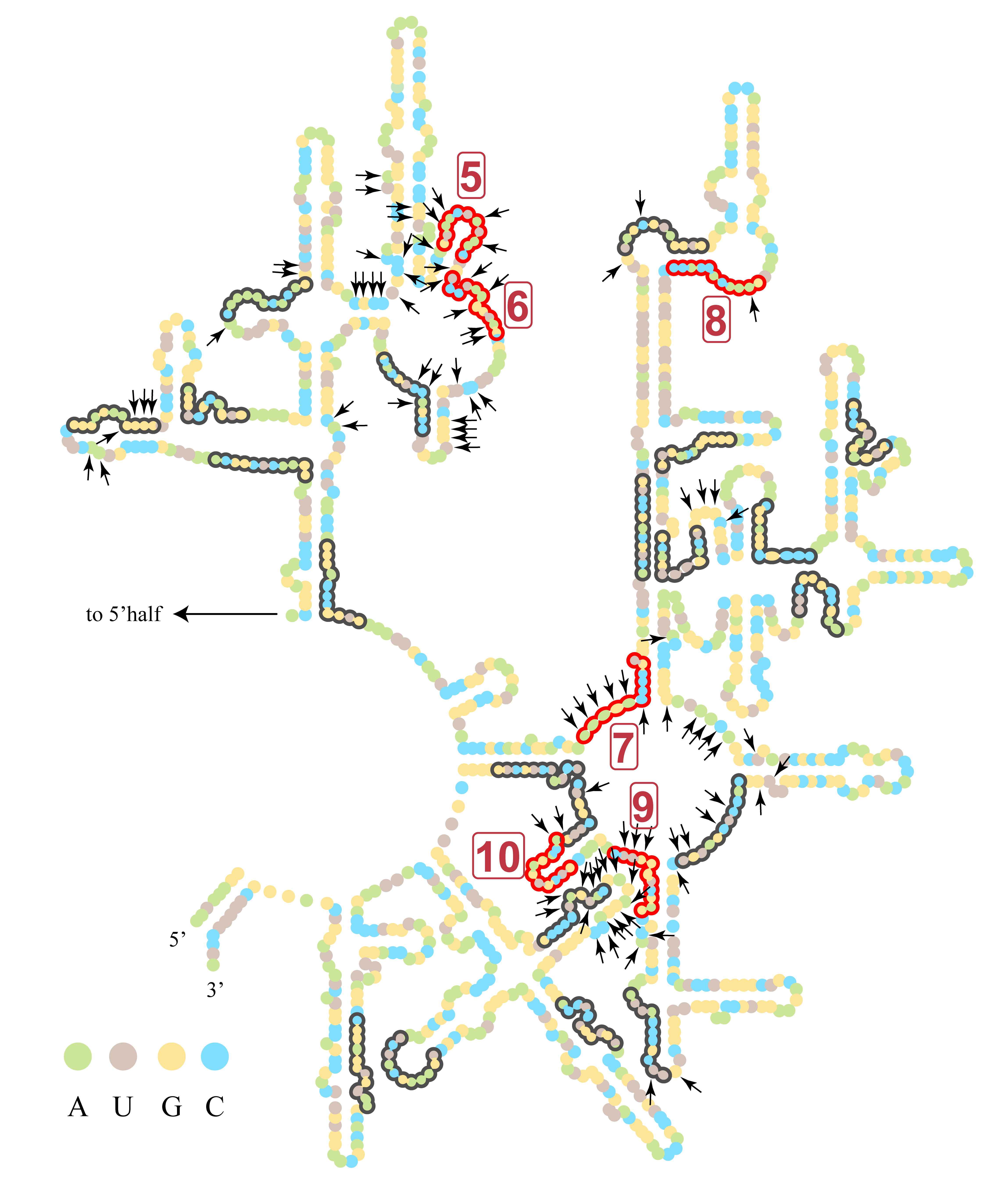

Supplement: Supplementary file 1 [file biology-11-00837-s001.zip › Figure S5.tif]
